# Selective inhibition of EZH2 by ZLD1039 blocks H3K27methylation and leads to potent anti-tumor activity in breast cancer

**DOI:** 10.1038/srep20864

**Published:** 2016-02-12

**Authors:** Xuejiao Song, Tiantao Gao, Ningyu Wang, Qiang Feng, Xinyu You, Tinghong Ye, Qian Lei, Yongxia Zhu, Menghua Xiong, Yong Xia, Fangfang Yang, Yaojie Shi, Yuquan Wei, Lidan Zhang, Luoting Yu

**Affiliations:** 1 State Key Laboratory of Biotherapy and Cancer Center, West China Hospital, West China Medical School, Sichuan University, Chengdu 610041, China; 2 College of Chemical Engineering, Sichuan University, Chengdu, Sichuan 610065, China; 3 College of Chemistry and Life Science, Chengdu Normal University, Chengdu 611130, China

## Abstract

Enhancer of zeste homolog 2 (EZH2) is a candidate oncogenic driver due to its prevalent overexpression and aberrant repression of tumor suppressor genes in diverse cancers. Therefore, blocking EZH2 enzyme activity may present a valid therapeutic strategy for the treatment of cancers with EZH2 overexpression including breast cancers. Here, we described ZLD1039 a potent, highly selective, and orally bioavailable small molecule inhibitor of EZH2, which inhibited breast tumor growth and metastasis. ZLD1039 considerably inhibited EZH2 methyltransferase activity with nanomolar potency, decreased global histone-3 lysine-27 (H3K27) methylation, and reactivated silenced tumor suppressors connected to increased survival of patients with breast cancer. Comparable to conditional silencing of EZH2, its inhibition by ZLD1039 decreased cell proliferation, cell cycle arrest, and induced apoptosis. Comparably, treatment of xenograft-bearing mice with ZLD1039 led to tumor growth regression and metastasis inhibition. These data confirmed the dependency of breast cancer progression on EZH2 activity and the usefulness of ZLD1039 as a promising treatment for breast cancer.

In eukaryotes, the posttranslational modifications of core histones play crucial roles in modulating chromatin and, thereby, control entire gene transcription processes. Among the various histone modifications, methylation events at lysine and arginine residues catalysed by histone methyltransferases (HMTs)^1^, have attracted intense attention recently because of their association with diseases and druggability^2^. Therefore, HMTs constitute an interesting target class for cancer therapy^2^. In addition, numerous selective small molecule HMT inhibitors have been discovered to exhibit anti-proliferative activity in different tumor types harbouring genetic alterations in HMTs^3^,^4^,^5^.

Histone methyltransferase EZH2, the catalytic core protein of the polycomb repressive complex (PRC2)^6^, predominantly catalyses the trimethylation of histone-3 lysine-27 (H3K27), which leads to the transcriptional silencing of target genes involved in cell cycle regulation, differentiation, and proliferation^7^. Accumulating evidence implicates EZH2 in a wide variety of cancers by mutation, amplification, or over-expression, or a combination of these processes, which makes it an attractive anticancer drug target^3^,^8^,^9^.

Breast cancer is ranked as the second leading cause of cancer deaths among women worldwide^10^. Although substantial progress has been made in breast cancer detection and treatment, survival tends to be unsatisfactory^4^,^11^,^12^. Therefore, new treatments for breast cancer are urgently needed. The discovery of genetic alterations in HMTs that support the importance of epigenetic deregulation in breast cancer has undoubtedly made HMTs, including EZH2, the focus of much attention. The overexpression of EZH2 has been found to be a promising biomarker of aggressive breast cancers with poor prognosis and highly correlated with invasiveness as well as increased proliferation rates of breast carcinomas^13^,^14^. These studies proposed that abnormally elevated EZH2 can activate several signalling pathways such as the retinoblastoma protein-E2 promoter binding factor (pRB-E2F), phosphoinositide 3-kinase (PI3K)/Akt, and oestrogen receptor pathways by silencing the related tumor suppressor genes and promoting proliferation^15^,^16^,^17^.

Several studies have demonstrated that EZH2 knockdown decreased cell proliferation *in vitro* and significantly decreased breast xenograft growth *in vivo*^18^,^19^,^20^. GSK343, the potent and selective inhibitor of EZH2, was recently discovered and reported to selectively reduce trimethylated H3K27 (H3K27me3) and inhibit breast cancer cell proliferation in cell-based studies^21^. However, no orally bioavailable inhibitors of EZH2 have been used in breast cancer *in vivo* models. Here, we developed ZLD1039, a novel potent and selective inhibitor of EZH2^22^, which inhibited the methyltransferase activity of EZH2 with high selectivity across an HMT panel. ZLD1039-treated breast cancer cells exhibited decreased H3K27me3 and H3K27me2 without changes in EZH2 and other H3 methylation markers. We found that inhibition of EZH2 by ZLD1039 in breast cancer cells upregulated several cancer suppressor genes and decreased cell proliferation and cell cycle arrest, as well as induced apoptosis. Moreover, ZLD1039-treated mice showed a considerable decreased in breast xenograft growth and metastasis in experimental breast tumor models. These data as well as the low toxicity of this compound, provide a basis for the further investigation of ZLD1039 as a potential treatment for breast cancer.

## Results

### Biochemical characterization of ZLD1039 as a potent and selective inhibitor of EZH2

**Figure 1:**
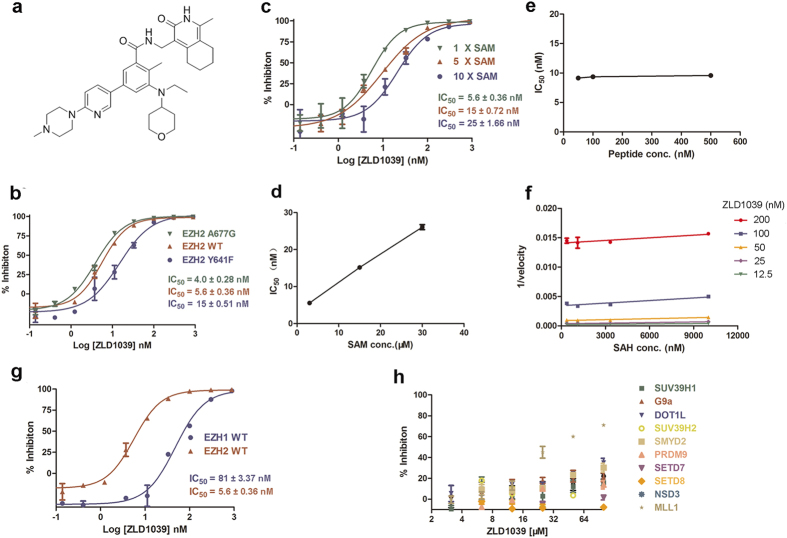
Characterization of a potent, selective EZH2 small molecule inhibitor. (**a**) Chemical structure of ZLD1039. (**b**) Potency of ZLD1039 against wild-type and mutant EZH2. IC_50_ values were calculated from 10 point dose response. All data are mean ± SD of triplicate experiments. (**c**,**d**) S-Adenosylmethionine (SAM) competition inhibition of ZLD1039. Activity assays were carried out with either standard assay, 5×SAM or 10 × SAM. The ~4-fold shift of IC_50_ values at higher SAM was consistent with ZLD1039 being SAM-competitive. Data are derived from 10 point dose response. All data are mean ± SD of triplicates. (**e**) Plot of IC_50_ values of ZLD1039 under the conditions of various peptide (H3[21–24]) concentrations. The IC_50_ was unaffected as the concentration of peptide was increased. All data points are mean of triplicates ± SD. (**f**) Assays were performed by combining various concentrations of SAH and ZLD1039 and yielded parallel lines in a plot of 1/velocity as a function of SAH concentration for different concentrations of ZLD1039 tested. All data points are mean of triplicates ± SD. (**g**,**h**) Selectivity of ZLD1039 was tested against a panel of HMTs, including EZH1, SETD7, SUV39H1, G9a, DOT1L, SUV39H2, SMYD2, PRDM9, SETD8, NSD3 and MLL1. All enzyme assays were carried out under balance conditions as six-point dose response. All data points are mean ± SD of triplicates. HMTs, histone methyltransferases; SETD7, SET domain containing lysine methyltransferase 7; SUV39H1, suppressor of variegation 3–9 homolog 1; DOT1L, DOT1-like histone H3K79 methyltransferase; SMYD2, SET and MYND domain containing 2; PRDM9, positive regulatory domain containing zinc finger protein 9; NSD3, nuclear receptor SET domain-containing 3; MLL1, mixed-lineage leukemia 1.

Recently, several EZH2 small molecule inhibitors were discovered, and these compounds are structurally related to the pyridones^23^,^24^,^25^,^26^. We previously designed and synthesized numerous compounds based on a pyridone-containing chemical scaffold^22^ using classical medicinal chemistry to identify EZH2 small molecule inhibitors, and ZLD1039 (Fig. 1a) was selected as the lead compound for its desirable potency and physicochemical properties. In biochemical assays using reconstituted PRC2, ZLD1039 showed potent and concentration-dependent inhibition of PRC2 enzymatic activity against EZH2 wild-type as well as Y641F, and A677G mutant enzymes with half-maximal inhibitory concentration (IC_50_) values of 5.6 ± 0.36, 15 ± 0.51, and 4.0 ± 0.28 nM, respectively (Fig. 1b). To determine the mode of inhibition (MOI) of ZLD1039, S-adenosylmethionine (SAM) and H3 peptide competition experiments were performed under saturated substrate peptide conditions. Consistent with competitive inhibition, the IC_50_ for ZLD1039 inhibition of EZH2 increased linearly with increasing concentration of SAM (Fig. 1c,d). However, the IC_50_ was independent of the peptide substrate concentration (Fig. 1e). These results suggest that ZLD1039 inhibited EZH2 competitively and non-competitive with SAM and the peptide substrates, respectively.

A previous study demonstrated that the enzymic catalytic reaction product S-adenosylhomocysteine (SAH) inhibited EZH2 in an SAM-competitive manner^27^. The overlapping binding sites for SAM and SAH have been confirmed by crystallographic analysis of the two ligands of PMTs^28^. To determine whether ZLD1039 bound in a mutually exclusive manner with SAH, the reaction velocity of SAH in the presence of several different ZLD1039 concentrations was detected. As shown in Fig. 1f, the data were best fit to parallel lines, which indicated the mutually exclusive binding of ZLD1039 and SAH.

The above data suggest that the SAM binding pocket of the EZH2 SET domain was the most plausible binding site of ZLD1039. However, definitive proof of binding within the SET domain of EZH2 requires structural confirmation using crystallographic methods. Recently, the intact PRC2 and EZH2 crystal structures were reported^29^. Future structural studies of the ZLD1039-EZH2 co-crystal structure may help to confirm the binding site for ZLD1039.

We also assessed the inhibitory effect of ZLD1039 against a panel of HMTs other than EZH2. ZLD1039 displayed a 14-fold selectivity for EZH2 over the closely related EZH1 and >10,000-fold selectivity over 10 other HMTs tested (Fig. 1g,h and Supplementary Table S3). Taken together, our biochemical results suggest that ZLD1039 is a potent and selective inhibitor of EZH2.

### Intracellular inhibition of H3K27 methylation

**Figure 2:**
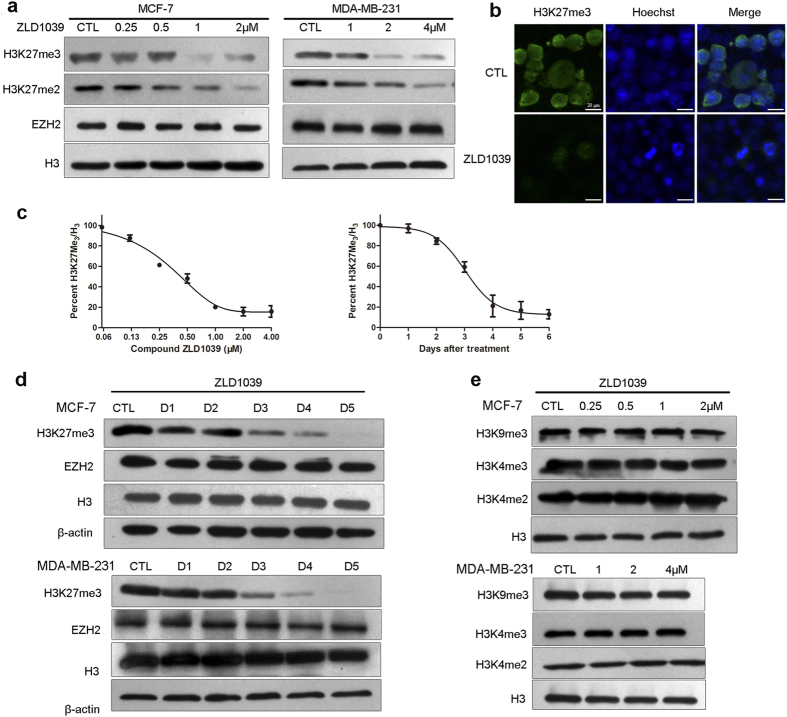
Effects of ZLD1039 on cellular global histone methylation. (**a**) Dose-dependent inhibition of cellular H3K27 methylation by 4-day ZLD1039 treatment of MCF-7 and MDA-MB-231 cells. H3K27me3, H3K27me2, EZH2, and H3 were detected using immunoblot. (**b**) H3K27me3 levels were analysed using immunofluorescence (IF) microscopy. MCF-7 cells were treated with 2 μM ZLD1039 for 3 days and Hoechst staining was used to illustrate similar cell numbers in both treatment groups. (**c**) ZLD1039 inhibited cellular H3K27me3 in MCF-7 cells time- and dose-dependently (ELISA assay). Data are mean ± SD of three independent experiments. (**d**) Time course of H3K27me3 inhibition by ZLD1039. MCF-7 and MDA-MB-231 cells were treated (2 and 4 μM, respectively) for indicated days. (**e**) ZLD1039 selectively inhibited cellular H3K27 methylation without other H3 methylation modifications in MCF-7 and MDA-MB-231 cells.

Previous studies using small interfering/short hairpin RNA (si/shRNA) to knock down EZH2 or other components of PRC2 showed that breast and prostate cancer cells were highly dependent on PRC2 for proliferation^18^,^19^,^20^. Therefore, we tested the ability of ZLD1039 to block methylation of H3K27 in breast cancer cells. ZLD1039 inhibited the H3K27me3 and H3K27me2 levels in MCF-7 and MDA-MB-231 cells in a dose-dependent manner (Fig. 2a,b) whereas the H3 and EZH2 were not affected. The results indicate that the reduction of H3K27me3 by ZLD1039 was due to the direct inhibition of EZH2 methyltransferase activity but not degradation of histone H3 or PRC2. Quantification of H3K27me3 using an enzyme-linked immunosorbent assay (ELISA) yielded an IC_50_ of 0.29 ± 0.09 μM in MCF-7cells in Fig. 2C. Similar results were obtained for other breast cancer and EZH2 mutant lymphoma cell lines (Supplementary Fig. S1).

A time-course study was performed in MCF-7 and MDA-MB-231 cells to elucidate the underlying mechanisms of the H3K27 methylation inhibition by ZLD1039. The H3K27me3 level was decreased 48 h after ZLD1039 treatment and reached the lowest level after 5 days (Fig. 2d). To test the specificity of ZLD1039, other histone methyl markers were examined and Fig. 2e shows that they were not changed, which was consistent with the biochemical assay data.

### Impact of ZLD1039 on breast cancer cell growth

**Figure 3:**
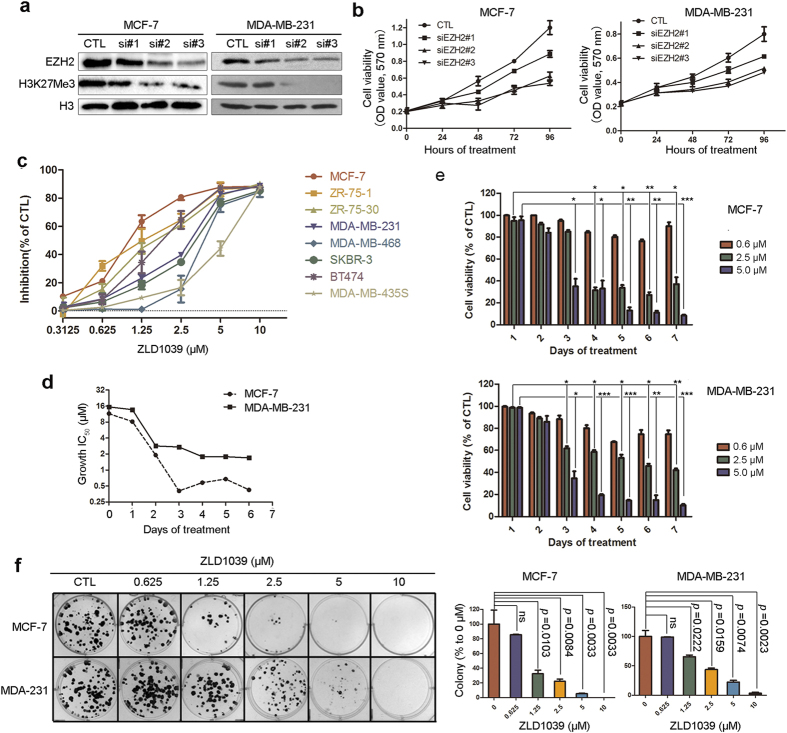
Antiproliferative activities of ZLD1039 against breast cancer cells *in vitro*. (**a**) Cells were transfected with EZH2 siRNA for 4 days. Levels of EZH2 expression were detected using immunoblots. (**b**) MCF-7 and MDA-MB-231 cells were transfected with EZH2 siRNA for 0, 1, 2, 3, and 4 days and cell viability was determined. Data are mean ± SD (n = 3). (**c**) Cells were treated with indicated agents for 4 days, and cell viability was measured using MTT assay. Results are expressed as mean ± SD of three independent experiments. (**d**) Potency of ZLD1039 against growth of MCF-7 and MDA-MB-231 cells over time represented as growth half-maximal inhibitory concentration (IC_50_, μM). (**e**) Proliferation of MCF-7 and MDA-MB-231 cells treated with various concentrations of ZLD1039 for indicated days. Values are mean ± SD (n = 3). ^*^P < 0.05, ^**^P *<* 0.01, and ^***^P *<* 0.001 compared with control. (**f**) Effects of ZLD1039 on MCF-7 and MDA-MB-231 cell colony formation after incubation for 14 days. Quantification is shown in right panel. Data are mean ± SD (n = 3). *P-*values for comparison of two groups were determined using two-tailed Student’s *t*-test; ns, not statistically significant.

To test whether downregulation of EZH2 contributes to the growth inhibition of breast cancer cells, three independent siRNAs specific to EZH2 were designed and transfected into MCF-7 and MDA-MB-231 cells. Two siRNAs, named siEZH2#2 and siEZH2#3, knocked down the EZH2 level by ~80% (Fig. 3a). Both siRNAs substantially reduced the viability of both cell lines (Fig. 3b), suggesting that the proliferation of breast cancer cells is dependent on EZH2.

To explore whether ZLD1039 treatment has a similar effect on cell viability as EZH2 knockdown, the *in vitro* antiproliferative activities of ZLD1039 against various breast cancer cell lines were examined using the 3–(4,5-dimethylthiazol–2-yl)-2,5-diphenyltetrazolium bromide (MTT) method. Eight human breast cancer cell lines with different genetic backgrounds namely ZR-75-1, ZR-75-30, MCF-7, MDA-MB-231, MDA-MB-468, SKBR-3, BT474, and MDA-MB-435S were chosen for this assay. The dose-response curves of ZLD1039 and IC_50_ values are shown in Fig. 3c and Supplementary Fig. S2b, respectively. ZLD1039 showed considerable dose-dependent antiproliferative activity against the tumor cell lines tested. Among the cell lines, MCF-7 and ZR-75-1 were the most sensitive to ZLD1039 with IC_50_ values of 0.99 ± 0.23 and 0.089 ± 0.019 μM, respectively. In addition, the time-course of the ZLD1039-induced effects was examined in detail in two of the sensitive cell lines. The MCF-7 cell line showed potent inhibition of cell proliferation after 2 days and reached the highest level after 4 days (Fig. 3d,e).

Then, we conducted a clonogenic assay to further evaluate the antiproliferative activity of ZLD1039. As shown in Fig. 3f and Supplementary Fig. S2c, both the size and the number of MCF-7, MDA-MB-231, and 4T1 clones were decreased even at low concentrations of ZLD1039.

### Induction of cell cycle arrest and apoptosis by ZLD1039 in breast cancer cells

**Figure 4:**
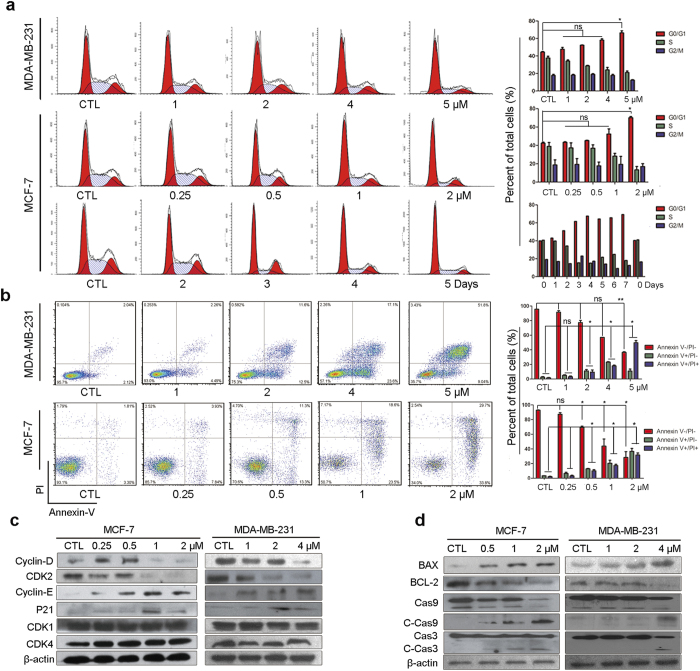
Induction of G0/G1 phase arrest and apoptosis by ZLD1039 treatment in breast cancer cells *in vitro*. (**a**) ZLD1039 induced G0/G1 phase arrest of MCF-7 and MDA-MB-231 cells. MDA-MB-231 cells (up) and MCF-7 cells (middle) were treated with indicated concentration of ZLD1039 for 4 days, and MCF-7 cells (down) were treated with ZLD1039 (2 μM) for indicated days, and then cell-cycle distribution was analysed by flow cytometry (FCM). Percentages of the cell cycle are presented (right). Values are mean ± SD (n = 3); ns, not statistically significant; ^*^P < 0.05 among groups. (**b**) FCM analysis of cells stained with Annexin V- FITC/PI after treatment with various concentrations of ZLD1039 for 4 days. Quantified values of apoptosis are shown (right). Results are mean ± SD of three independent experiments; ns, not statistically significant; ^*^P < 0.05 and ^**^P < 0.01 among groups. (**c**,**d**) Cells were treated with various concentrations of ZLD1039 for 4 days and proteins were analysed using immunoblot analysis. Cas9, pro-caspase 9; C-Cas9, cleaved-caspase 9; Cas3, pro-caspase 3; C-Cas3, cleaved-caspase 3.

To understand the role of EZH2 in the inhibition of the proliferation of breast cancer cells, we first performed a cell-cycle analysis. In MCF-7 cells treated with 2 μmol/L ZLD1039 for 1–7 days, a substantial accumulation of cells in G0/G1phase was detected (Fig. 4a). The percentage of cells in the G0/G1phase increased 2 days after ZLD1039 treatment and reached the highest level after 4 days. Then, MCF-7 and MDA-MB-231 cells were treated with increasing concentrations of ZLD1039 up to 2 and 5 μmol/L, respectively for 4 days and similar results were observed. Cell cycle progression is regulated by various cyclins and related cyclin-dependent kinases. Therefore, we determined the expression of cyclin-dependent kinases (CDKs) and cyclins in ZLD1039-treated cells. As shown in Fig. 4c, cyclin D and CDK2 protein levels were decreased after 4 days of treatment with ZLD1039, whereas cyclin E was increased and the other cyclins determined were not affected. These results were consistent with the accumulation of G0/G1 population in the fluorescence-activated cell sorting (FACS) analysis. Next, we investigated the induction of apoptosis by ZLD1039 treatment using Annexin V/propidium iodide (PI) staining. Exposure of MCF-7 and MDA-MB-231 cells to ZLD1039 for 4 days significantly increased the Annexin V positive cell population suggesting an increase in apoptosis (Fig. 4b). To further confirm the induction of apoptosis by ZLD1039, we examined the expression of apoptosis-related proteins in ZLD1039-treated cells (Fig. 4d). Indeed, the B-lymphoma 2 (Bcl-2) family members, Bcl-2 and Bcl-2 associated X protein (BAX) were decreased and increased, respectively. Furthermore, cleaved-caspase 3 and cleaved-caspase 9 levels were elevated with increasing concentrations (Fig. 4d). Taken together, these results indicate that inhibition of EZH2 by ZLD1039 may considerable block cell cycle progression and induce apoptosis in breast cancer cells.

### Identification of PRC2-repressed genes reactivated by ZLD1039 in breast cancer cells

**Figure 5:**
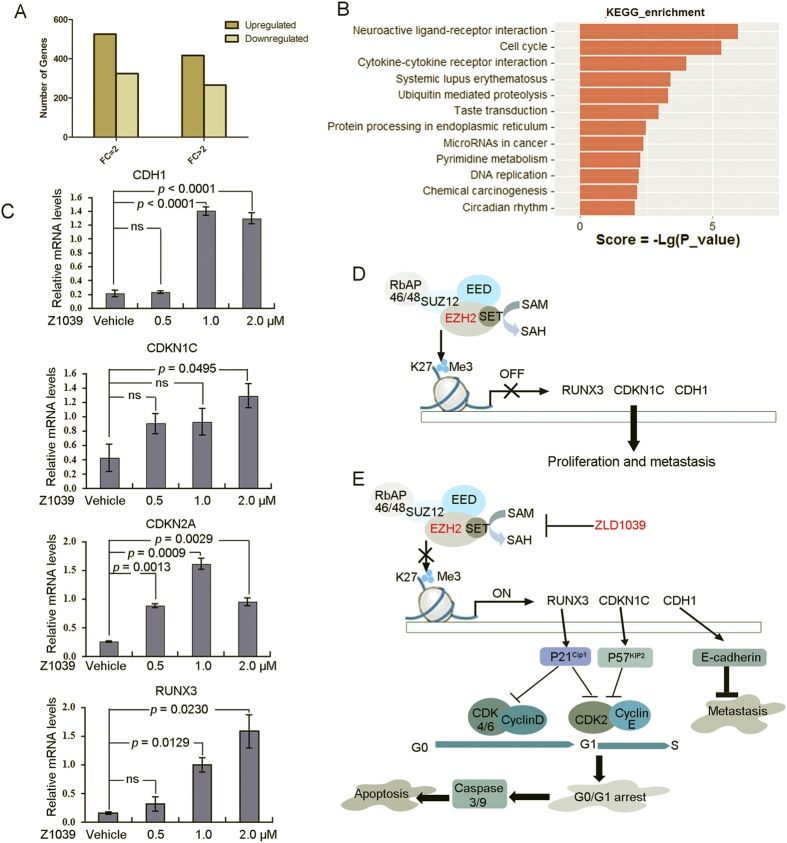
ZLD1039 induced transcription activation in MCF-7 cells. (**A**) Gene-expression changes in MCF-7 cells after ZLD1039 (1.5 μM for 3 days) treatment. Changes in gene expression were evaluated using fold-change >2 and *P* < 0.05. (**B**) KEGG analysis of expression microarray assays of PRC2 in MCF-7 cells, categorized by molecular function (MF). Top 12 pathways with the highest significance in KEGG analysis were listed. KEGG, Kyoto Encyclopedia of Genes and Genomes. (**C**) MCF-7 cells were incubated with indicated concentration of ZLD1039 for 3 days. Gene expression was determined using qRT-PCR and expressed relative to control of each time point. Data are mean ± SD of three independent experiments. *P-*values for comparison of two groups were determined using two-tailed Student’s *t*-test; ns, not statistically. (**D**,**E**) Proposed signalling pathways of ZLD1039-induced G0/G1 arrest, apoptosis, and antimetastatic activity in breast cancer cells.

EZH2 is associated with transcriptional repression in breast cancer and, therefore, we examined the ZLD1039-induced gene-expression changes in MCF-7 cells using gene expression arrays (Supplementary Fig. S3 and Table S5). Not surprisingly, more genes were up-regulated than down-regulated after ZLD1039 treatment (Fig. 5A), which was consistent with EZH2’s role in transcriptional repression. To further delineate the pathways that were regulated by ZLD1039 through EZH2 inhibition, we performed a Kyoto Encyclopedia of Genes and Genomes (KEGG) analysis of the microarray data (*P* < 0.05, Fig. 5B). Cell cycle and cytokine-cytokine receptor interaction-associated genes showed the highest significance in the microarray KEGG analysis, consistent with our data on cell cycle and apoptosis. We further selected six genes associated with cell cycle and metastasis that were up-regulated by ZLD1039 and validated their expression using quantitative real-time reverse transcription-polymerase chain reaction (qRT-PCR, Fig. 5C). Among the genes, *RUNX3*, *CDKN1C*, and *CDH1* were considerable up-regulated. As shown in Fig. 4c, the *RUNX3* gene downstream protein p21 was recovered while CDK2 and cyclin D were decreased. Collectively, these results suggest that ZLD1039 inhibited EZH2 enzyme activity, up-regulated CDK inhibition, lead to cell cycle arrest and apoptosis (Fig. 5D,E).

### Anti-tumor activity of ZLD1039 in breast xenograft animal models

**Figure 6:**
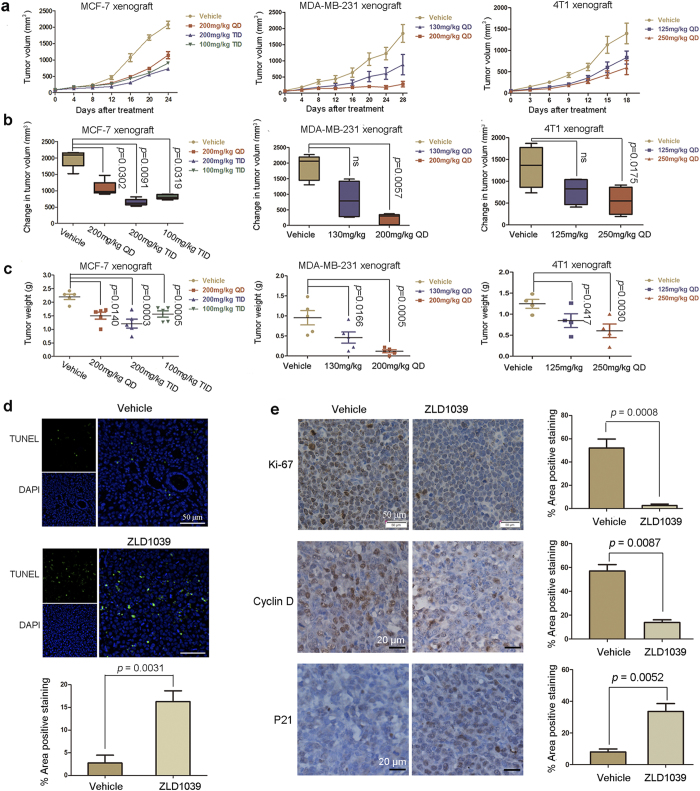
Antitumor efficacy of ZLD1039 *in vivo*. (**a**) Tumor regression of MCF-7, MDA-MB-231, or 4T1 tumor xenografts in mice treated with different dosing schedules of ZLD1039 three times a day (TID) or once a day (QD). Data are tumor volume mean ± SEM (n = 5). (**b**) Quantitative analysis of tumor volume change on final study day. Boxes display lower (25th) and upper (75th) quartiles with a line at the median; whiskers extend from minimum to maximum observation. *P-*values were determined using two-tailed Student’s *t*-test; ns, not significant. (**c**) Represented weight of tumors from mice in different groups. Data are mean ± SEM. *P-*values comparing two groups were determined using two-tailed Student’s *t*-test. (**d**) After 14 days treatment, MCF-7 tumors treated with 200 mg/kg ZLD1039 or vehicle were examined using terminal deoxynucleotidyl transferase (TdT) dUTP nick-end labelling (TUNEL) assay (n = 3). TUNEL-positive cells were counted in four high power fields/slide, and data were summarized as a percentage of positive cells. Data are mean ± SD. *P-*values were determined using two-tailed Student’s *t*-test. (**e**) Tumor tissues from MCF-7 xenografts treated with vehicle or ZLD1039 (200 mg/kg) for 14 days were immunohistochemically analysed with anti-Ki67, anti-cyclin D, and anti-P21 antibodies (n = 3). Representative images and quantitative analysis of percentage of positive staining are shown. *P-*values for comparing two groups were determined using two-tailed Student’s *t*-test.

To evaluate the antitumor activity of ZLD1039 *in vivo*, three breast tumor xenograft models namely MCF-7, MDA-MB-231, and 4T1 cell lines models were used in this study. In all the models, ZLD1039 markedly induced tumor growth inhibition (TGI) in a dose-dependent manner (Fig. 6a,b,c and Supplementary Fig. S4a). TGI levels of 86.1 and 58.6% were observed at 200 and 250 mg/kg in the MDA-MB-231 and 4T1 cell models, respectively. In the MCF-7 cell model, the highest dosage of 200 mg∙kg^−1^∙day^−1^ administered in 3 divided doses showed the maximum TGI of 67.5%. Furthermore, the 100 mg∙kg^−1^∙day^−1^ administered in 3 divided doses induced a higher TGI than 200 mg∙kg^−1^∙day^−1^ did. The results may due to the pharmacokinetic properties of ZLD1039 (Supplementary Table S4). Following oral administration, ZLD1039 was rapidly absorbed with maximal concentrations achieved 15 min after dosing. Furthermore, the mean residence time was 3.29 h with an elimination half-life (t_1/2_) of 4.36 h in rats. Moreover, ZLD1039 was well tolerated with only slight effects on the body weight of all the treatment groups (Supplementary Fig. S4b), which was confirmed by the results from sub-acute toxicity study in mice (Supplementary Fig. S5).

To examine the effects of ZLD1039 on cell cycle and apoptosis *in vivo*, MCF-7 tumors were harvested on day 14 after treatment with ZLD1039 for immunohistochemistry and terminal deoxynucleotidyl transferase (TdT) dUTP nick-end labelling (TUNEL) assays. As depicted in Fig. 6d,e, ZLD1039 potently inhibited the expression of cyclin D (from 57 ± 5.4 to 13.8 ± 2.3%) and increased that of p21 (from 8 ± 1.9 to 33.6 ± 4.9%). In addition, there was a substantial decrease in tumor cell proliferation (Ki-67-positive cells, from 52 ± 7.7 to 2.6 ± 1.2%) as well as a clear increase in apoptosis (TUNEL- positive cells, from 2.7 ± 0.7 to 16.3 ± 2.4%) compared with tumors of the group treated with vehicle. Overall, these results showed that ZLD1039 regulated the expression of specific cell cycle-associated proteins *in vivo*, thereby blocking proliferation, and inducing apoptosis in human breast tumor xenograft models.

### Antimetastatic activities of ZLD1039 against breast cancer cells *in vitro* and *in vivo*

**Figure 7:**
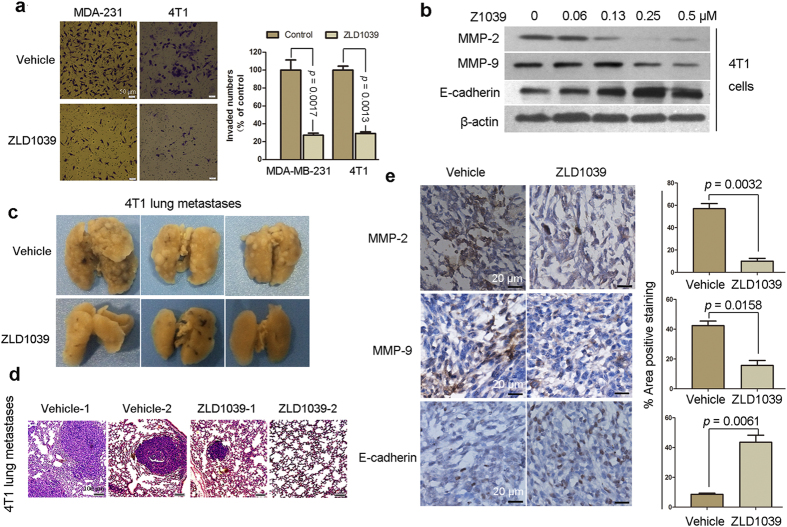
ZLD1039 inhibited tumor metastasis. (**a**) MDA-MB-231 and 4T1 cells were seeded in top chamber of transwell and treated with ZLD1039 (2 and 1 μM, respectively) for 24 h. Migrated cells were stained, photographed, and quantified. Results are mean ± SD of three independent experiments. *P-*values for comparing two groups were determined using two-tailed Student’s *t*-test. (**b**) 4T1 cells were treated with indicated concentration of ZLD1039 for 4 days and expression of proteins were detected using immunoblot. (**c**) Metastatic lung nodules were visualized to show antimetastatic activity of ZLD1039 (250 mg/kg) against 4T1 tumors after 18-day treatment. (**d**) Haematoxylin and eosin (H&E) staining of lung tissues was performed, and representative images are shown. (**e**) Tumors were harvested from 4T1 tumor-bearing mice after 18-day treatment with ZLD1039 (250 mg/kg) and expression of matrix metalloproteinase MMP-2, MMP-9, and E-cadherin was detected using immunohistochemical (IHC) analysis (n = 3). Representative images and quantitative analysis of percentage of positively stained cells are shown. *P-*values for comparing two groups were determined using two-tailed Student’s *t*-test.

Previous studies showed that EZH2 promotes migration and invasion of breast cancer cell lines^30^,^31^. Therefore, we further assessed the effect of ZLD1039 on tumor metastasis. MDA-MB-231 and 4T1 cells were used for the transwell assay to assess the drug effects on invasiveness because these cell lines possess metastatic ability. As shown in Fig. 7a, ZLD1039 potently blocked the invasion of the MDA-MB-231 and 4T1 cells with invasion inhibition rates of 72.6 ± 4.4 and 70.9 ± 2.6%, respectively. Furthermore, matrix metalloproteinase MMP-2, MMP-9, and E-cadherin, which are considered to be associated with cell migration and invasion were detected in 4T1 cells. As shown in Fig. 7b, MMP-2 and MMP-9 was decreased, whereas E-cadherin was increased after ZLD1039 treatment.

The 4T1 murine breast cancer cell line has a high metastatic potential and can spontaneously metastasize to the lung after inoculation in BALB/C female mice. Therefore, we investigated the effect of ZLD1039 on 4T1 lung metastasis. On day 18, the number and size of the metastatic lung nodules were markedly reduced in the ZLD1039-treated mice compared with the vehicle-treated mice (Fig. 7c). In addition, examination of the intact lungs using haematoxylin and eosin (H&E) staining confirmed that the number of micrometastatic nodules in the ZLD1039-treated group was fewer than that in the vehicle-treated group (Fig. 7d). Then, the expression of MMP-2, MMP-9, and E-cadherin was detected in 4T1 graft tumors treated with ZLD1039 for 18 days, using an immunohistochemical (IHC) assay. The results indicated that treatment with ZLD1039 substantially inhibited the expression of MMP-2 and MMP-9 and increased that of E-cadherin (from 57.1 ± 4.4 to 10.1 ± 2.4, 42.4 ± 3.1 to 15.7 ± 3.3, and 8.6 ± 0.7 to 43.6 ± 4.0%, respectively) *in vivo*, which was similar to the *in vitro* results (Fig. 7e). Moreover, the gene expression arrays revealed that *CDH1* was up-regulated by EZH2 inhibition following ZLD1039 treatment (Fig. 5C). Therefore, the antimetastatic activity of ZLD1039 may be due to the recovery of *CDH1* (E-cadherin), and associated with MMP-2 and MMP-9 (Fig. 5D,E).

## Discussion

The HMTs are an epigenetic enzyme class that play a paramount role in the regulation of gene transcription. The several genetic alterations discovered in the HMTs have been shown to drive specific human cancers^32^,^33^. The enzyme EZH2 is the catalytic subunit of the PRC2 and acts on HMT to catalyse the site-specific methylation of H3K27^34^. Importantly, PRC2 maintains the transcriptional repression of numerous genes with regulatory roles in cell development and differentiation^7^. Numerous studies suggest that up-regulation of H3K27me3 levels by EZH2 overexpression or mutation could silence tumor suppressors connected to promoting tumor growth and metastasis. Furthermore, this suggestion has attracted attention to this enzyme as a potential therapeutic target.

Overexpression of EZH2 is frequently detected in invasive and metastatic breast cancers, and associated with cancer progression or poor prognosis^13^,^14^. Notably, EZH2 appears to be not only a promising tumor biomarker but may itself contribute to tumor progression, similar to an oncogene. EZH2 disruption by si/shRNA, DZNep (an indirect and general inhibitor of methyltransferases), or both has decreased cell proliferation, induced apoptosis *in vitro*^18^,^19^,^20^, and significantly decreased breast xenograft growth *in vivo*^18^. In the present study, we showed that pharmacological inhibition of EZH2 using a novel EZH2 inhibitor ZLD1039, reduced cell proliferation, induced breast cancer cell apoptosis, and led to the regression of three established breast tumor xenografts in mice with excellent tolerance. Collectively, these data confirm the dependency of breast cancers on PRC2 activity and provide evidence that the pharmacological inhibition of EZH2 could provide a basis for its use as a therapeutic intervention in breast cancers.

Apoptosis is a programmed cell suicide process that is essential for the development and maintenance of tissue homeostasis and its dysfunction results in numerous types of malignancies in humans^35^,^36^. Recently, Zhou *et al.*^37^ showed that the mitochondrial-dependent apoptosis in cancer cells was significantly induced when EZH2 was depleted. In this study, a decreased level of Bcl-2 and increased level of BAX were observed in breast cancer cells after ZLD1039 treatment. Activation of caspase 9 and caspase 3 was also observed after the treatment. The changes in proteins related to the mitochondrial cell death pathway suggest that the apoptosis induction by ZLD1039 might be mediated by EZH2 disturbance as well as damage to the mitochondrial membrane.

Since EZH2 acts as a transcriptional repressor, inhibition of its activity restores genes expression including those of cell cycle inhibitors and tumor suppressors, leading to decreased cell proliferation. Here, we demonstrated that more genes were up-regulated than down-regulated after ZLD1039 treatment in the microarray analysis, which was consistent with EZH2′s role in transcriptional repression. In addition, inhibition of EZH2 by ZLD1039 restored the expression of cell cycle inhibitors and tumor suppressors. *RUNX3* is an established breast tumor suppressor, and it has been demonstrated that its down-regulation is controlled by H3K27me3 through EZH2^38^. In this study, the expression of *RUNX3* was recovered by ZLD1039, which led to the up-regulation of the downstream protein p21 (Fig. 4c). The *CDKN1C* has been reported to be targeted by EZH2-mediated H3K27me3 in breast cancer, which encodes the tumor suppressor p57 protein^39^. Following treatment with ZLD1039, the recovery of *CDKN1C* expression was observed. The downstream p21 and p57 are all CDK inhibitors, which lead to the reduction of CDK2, CDK4/6, and cyclin D and delay the G0/G1 cell-cycle transition. Here, we found that CDK2 and cyclin D decreased after ZLD1039 treatment, consistent with the G0/G1 arrest in cell-cycle analysis. Finally, *CDH1* encodes the metastasis-related protein E-cadherin and has been reported to be associated with H3K27me3 levels in the promoter^40^. ZLD1039 inhibited breast cancer metastasis in the present study likely by the suppression of *CDH1*. Therefore, the combined effects of ZLD1039-mediated EZH2 inhibition on several cancer pathways likely mediates the dramatic antitumor activity seen in the breast cancer models.

However, transcription suppression is the result of the interaction between EZH2 and other chromatin modifiers such as histone deacetylases and DNA methyltransferases^41^. Future studies are needed to determine if the target genes, including the tumor suppressors, can be sufficiently reactivated by decreasing the H3K27me3 level alone. Furthermore, the information may facilitate our understanding of how to develop and use the EZH2 inhibitor as a single agent or in combination with other inhibitors for breast cancer treatment.

In conclusion, we have characterized an EZH2 inhibitor, ZLD1039 that showed considerable antitumor activity both *in vitro* and *in vivo*. Combined with several molecules have been successfully applied in preclinical cancer models^23^,^24^,^25^,^26^ and are likely to be taken forward to clinical trails, the present data suggest that pharmacological inhibition of EZH2 enzymatic activity could provide a basis of for its use as a therapeutic intervention in non-Hodgkin lymphoma and solid tumors, which are dependent on EZH2 enzymatic activity and ZLD1039 is worth being optimized and develop as a therapeutic agent for clinical application.

## Methods

### Cell culture and reagent

The human breast cancer cell lines MCF-7, ZR-75-1, ZR-75-30, SKBR3, BT-474, MDA-MB-231, MDA-MB-468, and MDA-MB-435S were all acquired from the American Type Culture Collection (ATCC, Manassas, VA, USA). The cells were cultured in Dulbecco’s modified Eagle’s medium (DMEM) or Rosewell Park Memorial Institute (RPMI) 1640 media containing 10% foetal bovine serum (FBS) and 0.1% amikacin sulphate under humidified conditions with 5% CO_2_ at 37 °C. No further authentication was conducted for the tumor cell lines. ZLD1039 was synthesized at the State Key of Laboratory of Biotherapy (Sichuan University, Sichuan, China)^22^.

### Biochemical Assay

The enzyme levels of the histone methyltransferase panel were determined using the AlphaLISA immunodetection assay conducted using the enzyme profiler service provided by Shanghai ChemPartner (Shanghai, China). The values were further determined using an AlphaLISA methyltransferase assay kit (PerkinElmer, MA, USA) according to the manufacturer’s protocol.

### Immunofluorescence (IF), immunohistochemistry (IHC), and western blot (WB) analyses

The IF, IHC, and WB analyses were conducted according to standard protocols with minor modifications due to antibody optimization. The antibodies used are listed in the Supplementary Table S1.

### ELISA

Proteins isolated from cells that were treated with ZLD1039 for 4 days at different concentration, and different periods at 2 μM were placed into the ELISA plates, and the assays were performed using the ELISA Accessory kit (Dakewei Biotech, Shenzhen, China) according to the manufacturer’s protocol. The final reaction mixture absorbance was read at 450 nm using a SpectraMax M 5 microplate reader.

### Cell proliferation and colony formation assay

A variety of human breast cancer cell lines were treated with the indicated concentrations of ZLD1039 for 4 days, and cell viability was determined using an MTT (Sigma-Aldrich, St. Louis, MO, USA) assay. The IC_50_ values were calculated using the GraphPad Prism 5.01 software. For the colony formation assay, cells were treated with ZLD1039 for 14 days, fixed with methanol, and then stained with crystal violet. Then, the colonies with >50 cells were counted under an inverted microscope.

### Cell cycle and apoptosis analyses

For the cell cycle analyses, the ZLD1039-treated cells were fixed and stained with the PI staining solution for 10 min in the dark and then analysed using flow cytometry (FCM). The data were analysed using the Cell Quest and Flow Jo software. For the apoptosis assays, the cells were harvested and analysed using an Annexin V- fluorescein isothiocyanate (FITC) apoptosis detection kit (Roche, Indianapolis, IN, USA) according to the manufacturer’s protocol.

### Cell invasion assay

The invasion assays were conducted according to standard protocols. Briefly, the upper chambers of 24-well transwell plates (Millipore) were coated with matrigel diluted in serum-free medium (1:1) while the lower chambers were filled with 600 μl of medium with 10% FBS. After the matrigel polymerization, the cells in serum-free medium were placed in the upper chambers and treated with different concentrations of ZLD1039. After a 24-h incubation, the non-migrated cells were removed from the upper side of the filter, and the migrated cells were fixed with 4% paraformaldehyde and stained with 0.05% crystal violet. The migrated cells were photographed, and the cell in six random fields of each well were counted under a light microscope.

### Gene expression profiling, data processing, and further analyses

Gene expression profiling was conducted using a profiler service provided by Shanghai Biotechnology Corporation (Shanghai, China). The total RNA was purified from MCF-7 cells after dimethyl sulphoxide (DMSO) or ZLD1039 (1.5 μM) treatment for 3 days. Triplicate samples were collected and hybridized to the GeneChip PrimeView Human Gene Expression Array (Affymetrix). Differential gene expression was determined using the limma statistical package (http://www.bioconductor.org), and we conducted pairwise contrasts of ZLD1039-treated versus the control. Significant probe sets were filtered for detection using fold-change >2 and *P* < 0.05 (Student *t*-test). The differential gene expressions (gCTL_ vs. gZ1039-T_, *P* < 0.05) were used in the KEGG analysis.

### SiRNA transfection and qRT-PCR analysis

Cells were transfected with validated siRNA for EZH2 at a concentration of 100 nM using jetPRIME DNA and siRNA Transfection Reagent (Polyplus-transfection SA, NY, USA). The EZH2 gene target-specific siRNAs were purchased from Gene Pharma (Shanghai, China) and the targeting sequences are listed in Supplementary Table S2. For the qRT-PCR analyses of mRNAs, total RNA was isolated using TRIZOL and reverse transcribed to cDNA using the PrimeScript 1st Strand cDNA Synthesis kit. The qRT-PCR reactions were performed using SYBR Green technology and the PCR primers are listed in Supplementary Table S2.

### Subcutaneous xenograft models

All animal experiments were approved by the Institutional Animal Care and Treatment Committee of Sichuan University, China (Permit Number: 20140807, 20141009 and 20141030) and were carried out in accordance with the approved guidelines.

For the MCF-7 tumor xenograft model, BALB/c nude mice received 5 × 10^6^ cells (0.1 mL cell suspension) subcutaneously on the right flank. When tumors had reached an average volume of 100 mm^3^, the mice were randomly divided into four groups (n = 5). ZLD1039 or the vehicle consisting of 12.5% each of polyoxyethylenated castor oil (EL) and ethanol (ETOH) in normal saline (NS) was administered at the indicated doses three times daily every 8 h or once a day for 24 days by oral gavage.

The MDA-MB-231 donor tumors were first prepared by subcutaneously implanting 1 × 10^7^ cells (0.1 mL cell suspension) into the right flank of BALB/c nude mice. The donor tumors were then dissected into fragments of approximate 20 mm^3^ and subsequently subcutaneously implanted into the BALB/c nude mice. After implantation, mice with tumors ranging 70–120 mm^3^ were selected and randomized into groups for the efficacy study (n = 5). ZLD1039 or the vehicle was administered once a day for 28 days by oral gavage.

The 4T1 breast cancer cells have a high metastatic potential and can spontaneously metastasize to the lung as early as 2 weeks after inoculation in mice^42^. In this study, 5 × 10^5^ 4T1 cells (0.1 mL cell suspension) were injected subcutaneously into the right flank of BALB/c female mice. About 4 days after tumor cell inoculation, the injection sites exhibited tumors, and after about 7 days, the tumor-bearing mice were randomized into three groups (n = 5). ZLD1039 or vehicle was administered once a day for 18 days by oral gavage. For all subcutaneous xenograft models, the tumor volumes and body weight were assessed every 3 days. Tumor volume was calculated using the following formula: tumor volume = length × width^2^ × 0.55.

### Pharmacokinetic analyses and sub-acute toxicity test

For the pharmacokinetic analysis, blood from Sprague-Dawley rats dosed with ZLD1039 was collected in pre-chilled K_2_-ethylenediaminetetraacetic acid (EDTA) tubes at pre-specified time intervals. The plasma samples were isolated by centrifugation, and the concentrations of ZLD1039 were determined using liquid chromatography-tandem mass spectrometry (LC-MS/MS). For the sub-acute toxicity test, the clinical symptoms of the BALB/C mice orally administered 2 g/kg ZLD1039 were observed. Blood was collected and subjected to haematological and serum biochemistry analyses using the Hitachi 7200 Blood Chemistry Analyzer and a Nihon Kohden MEK-5216K Automatic Hematology Analyzer. The main organs of the mice were H&E stained for pathology examination.

### Statistical analyses

The means and group differences of the data were statistically analysed using the Student’s *t*-test using the GraphPad Prism 5.01 software. A *P* < 0.05 was considered statistically significant.

## Additional Information

**How to cite this article**: Song, X. *et al.* Selective inhibition of EZH2 by ZLD1039 blocks H3K27methylation and leads to potent anti-tumor activity in breast cancer. *Sci. Rep.* **6**, 20864; doi: 10.1038/srep20864 (2016).

## Supplementary Material

Supplementary Information
