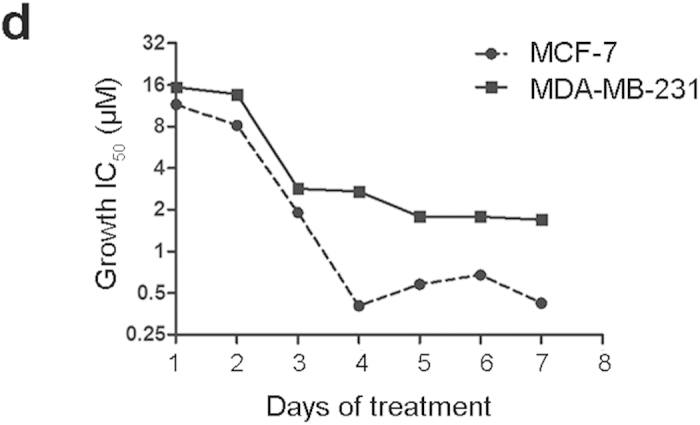# Corrigendum: Selective inhibition of EZH2 by ZLD1039 blocks H3K27methylation and leads to potent anti-tumor activity in breast cancer

**DOI:** 10.1038/srep24893

**Published:** 2016-04-29

**Authors:** Xuejiao Song, Tiantao Gao, Ningyu Wang, Qiang Feng, Xinyu You, Tinghong Ye, Qian Lei, Yongxia Zhu, Menghua Xiong, Yong Xia, Fangfang Yang, Yaojie Shi, Yuquan Wei, Lidan Zhang, Luoting Yu

Scientific Reports 6: Article number: 2086410.1038/srep20864; Published online: 02122016; Updated: 04292016

This Article contains typographical errors.

In the Results section under subheading ‘Impact of ZLD1039 on breast cancer cell growth’,

“Among the cell lines, MCF-7 and ZR-75-1 were the most sensitive to ZLD1039 with IC_50_ values of 0.99 ± 0.23 and 0.089 ± 0.019 μM, respectively”.

should read:

“Among the cell lines, MCF-7 and ZR-75-1 were the most sensitive to ZLD1039 with IC_50_ values of 0.99 ± 0.23 and 1.089 ± 0.019 μM, respectively”.

In Figure 3d, the x-axis ‘1–8 days of treatment’ was incorrectly given as ‘0–7 days of treatment’. The correct Figure 3d appears below as Fig. 1

## Figures and Tables

**Figure 1:**