# Supplementary material for: Selective inhibition of EZH2 by ZLD1039 blocks H3K27methylation and leads to potent anti-tumor activity in breast cancer
Source: Sci Rep. 2016 Feb 12;6:20864. doi: 10.1038/srep20864 (PMC4751454; doi:10.1038/srep20864)
Supplement: Supplementary Information [file srep20864-s1.pdf]

Supplementary data

Selective inhibition of EZH2 by ZLD1039 blocks H3K27methylation and leads to potent anti-tumor activity in breast cancer

Xuejiao Song[1,+], Lidan Zhang[1,+], Tiantao Gao[1], Ningyu Wang[1], Qiang Feng[1,3], Xinyu You[2], Tinghong Ye[1], Qian Lei[1], Yongxia Zhu[1], Menghua Xiong[1], Yong Xia[1], Fangfang Yang[1], Yaojie Shi[1], Yuquan Wei[1] and Luoting Yu[1,*]

[1]State Key Laboratory of Biotherapy and Cancer Center, West China Hospital, West China Medical School, Sichuan University, Chengdu 610041, China

[2]College of Chemical Engineering, Sichuan University, Chengdu, Sichuan 610065, China

[3]College of Chemistry and Life Science, Chengdu Normal University, Chengdu 611130, China

*Corresponding authors.

Address: State Key Laboratory of Biotherapy and Cancer Center, West China Hospital, West China Medical School, Sichuan University, 17 #3rd Section, Ren Min South Road, Chengdu 610041, China.

Tel.: +86-28-8550-3817;

Fax: +86-28-8516-4060.

E-mail: yuluot@scu.edu.cn (L.-T. Yu)

[+]These authors contributed equally to this work

Supplementary figures and legends

Supplementary Figure S1

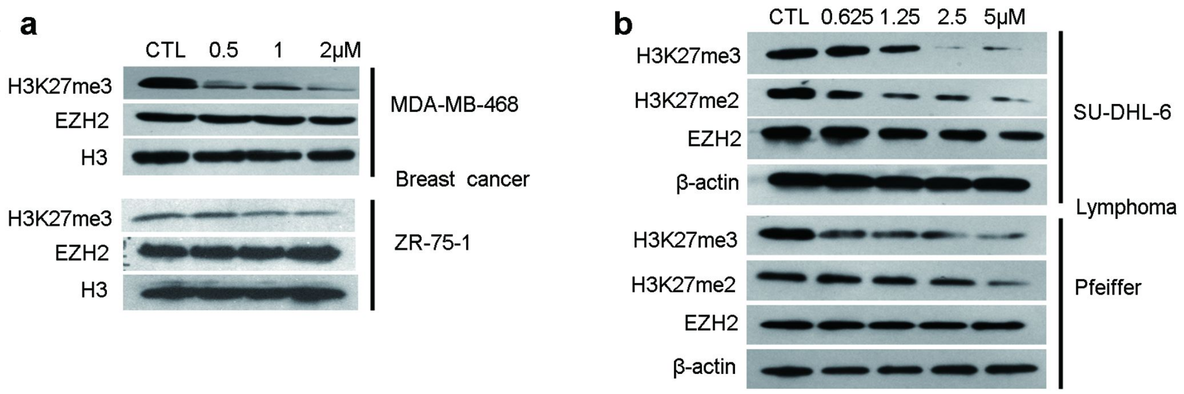

Supplementary Figure S1. Effects of ZLD1039 on cellular global histone methylation in breast cancer and lymphoma cells. (**a**) Dose-dependent inhibition of cellular H3K27 methylation by ZLD1039 for 4 days in ZR-75-1 and MDA-MB-468 cells. H3K27me3, EZH2, and H3 were detected by immunoblot. (**b**) ZLD1039 inhibited cellular H3K27 methylation in SU-DHL-6 and Pfeiffer cells in dose-dependent manner (4 days). H3K27me3, H3K27me2, EZH2, and H3 were detected by immunoblot.

Supplementary Figure S2

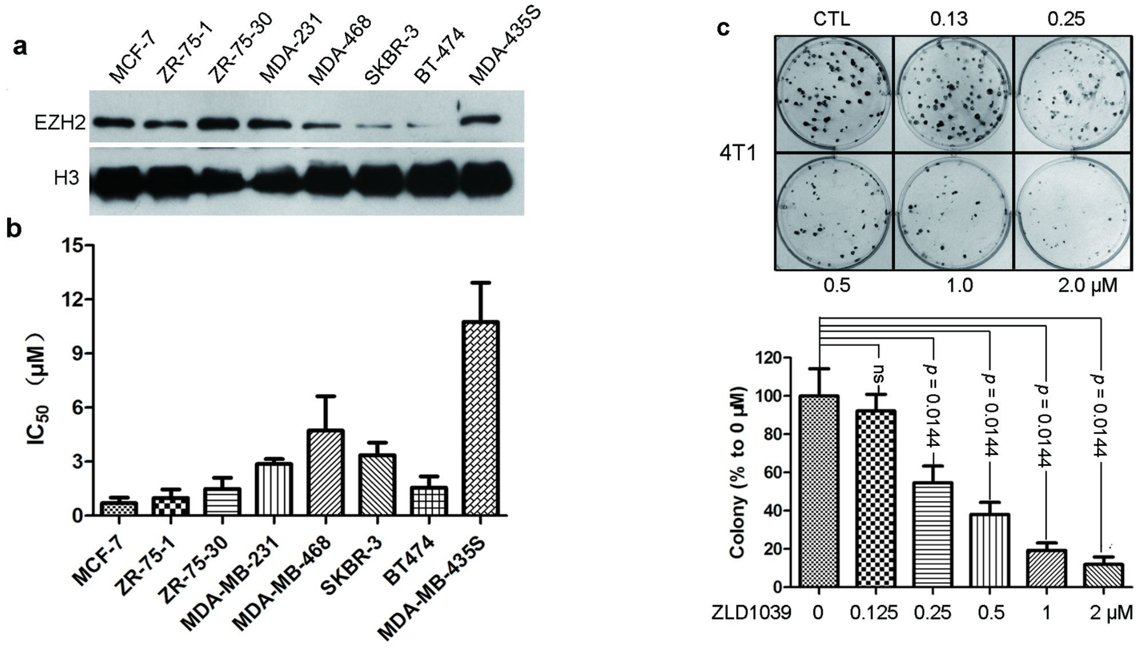

Supplementary Figure S2. Growth inhibition of breast tumor cell lines after treatment with ZLD1039. (**a**) The expression level of EZH2 in several breast cancer cell lines. (**b**) Cells were treated with ZLD1039 for 4days and cell viability was determined by MTT assay, represented as growth IC_{50} (μM). Results are expressed as mean ± SD of three independent experiments. (**c**) The effects of ZLD1039 on 4T1 cell colony formation after incubation for 14 days and quantification is shown in the down panel. Columns, mean; bars, SD; n=3. *P* values for comparison of two groups were determined by 2-tailed Student's *t*-test.

Supplementary Figure S3

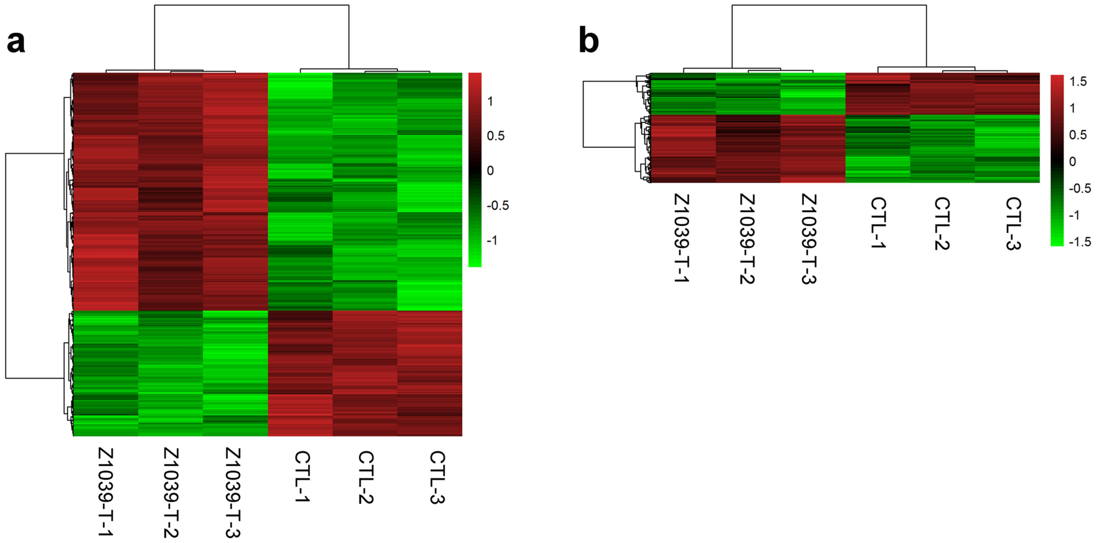

Supplementary Figure S3. Gene expression array heat maps. Heat maps of differently expressed genes in MCF-7 cells after EZH2 depletion by ZLD1039 treatment for 3 days (2μM) showing normalized expression value of three biological replicates. Changes in gene expression were filtered by fold-change > 2, P value < 0.05 (**a**) and fold-change > 2, P value < 0.01 (**b**).

Supplementary Figure S4

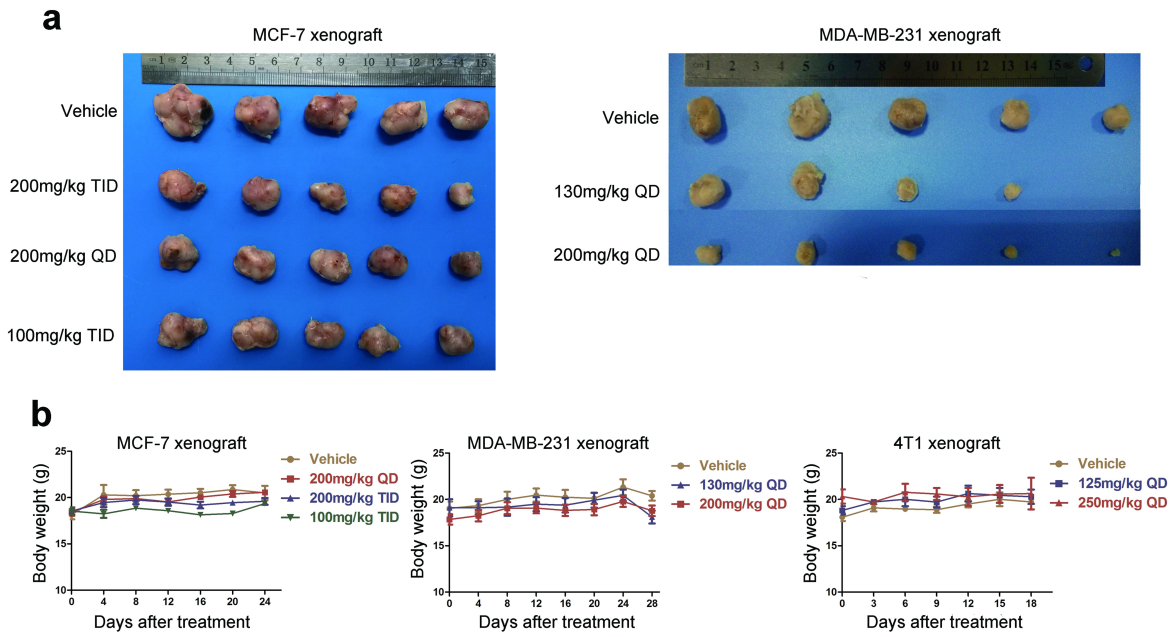

Supplementary Figure S4. Antitumor efficacy of ZLD1039 in vivo. (**a**) Representative photographs of subcutaneous tumors in each group of MCF-7 xenograft model (left) and MDA-MB-231 xenograft model (right). (**b**) Body weight of mice bearing breast tumor xenografts. There were no significant differences between treatment groups and vehicle group during the treatment cycle. Points, mean value; bars, SD.

Supplementary Figure S5

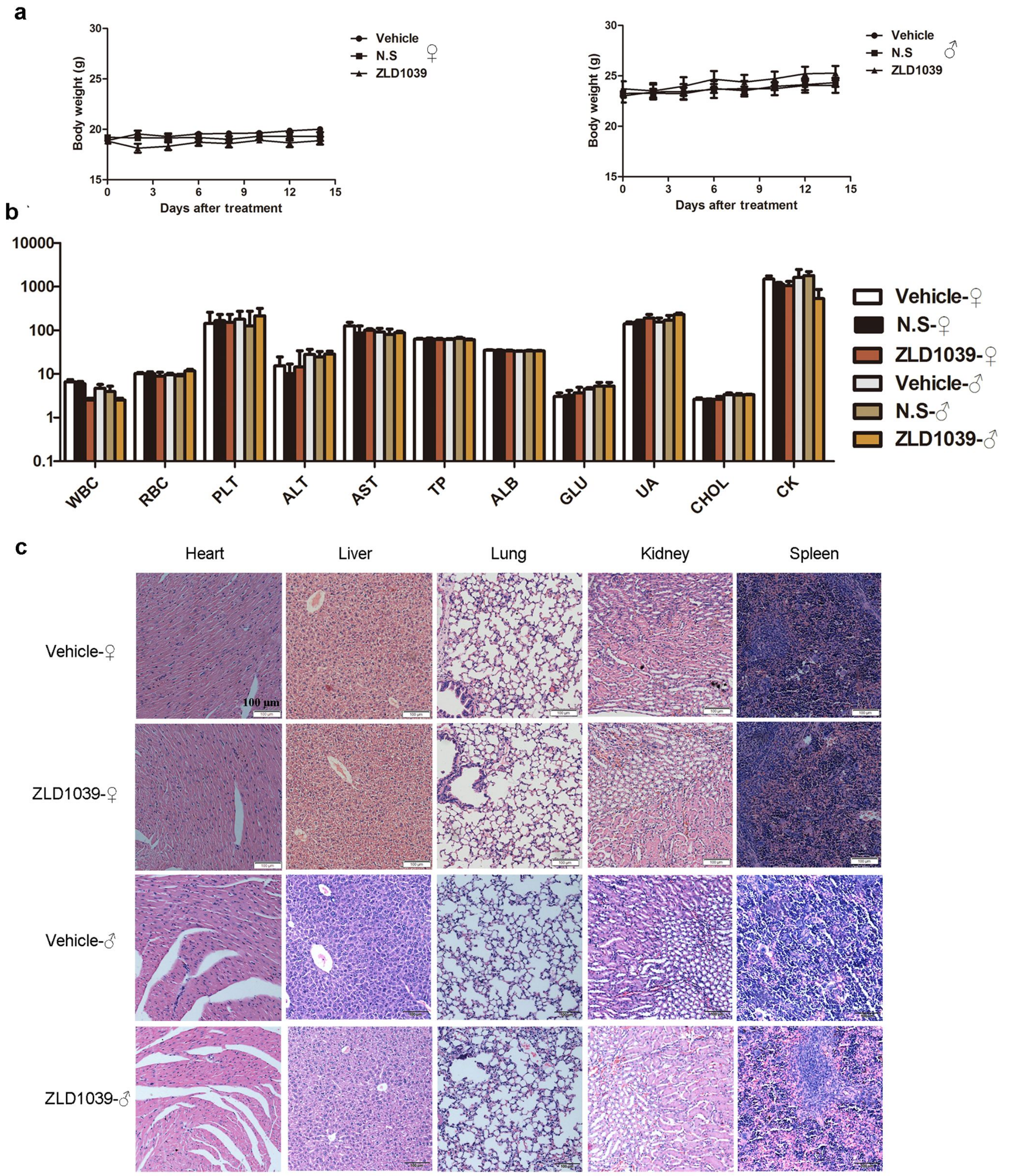

Supplementary Figure S5. Safety profile of ZLD1039 in BALB/C mice. (**a**) Data showing average body weight changes in the vehicle (n = 6) and ZLD1039 (2g/kg) treated mice (n = 6). Points, mean value; bars, SD. (**b**) Hematological and serum biochemical values of mice at day 14 (n = 6) for both vehicle and treated. Units of the parameters are as follows. WBC, PLT, 10^9/L; RBC, 10^{12}/L; TP, ALB, g/L; ALT, AST, CK, U/L; UA, GLU, uM; CHOL, mM. (**c**) ZLD1039 did not cause obvious pathologic abnormalities in normal tissues. H&E staining of

paraffin-embedded sections of the heart, liver, spleen, lung and kidney. Representative images of each group are shown.

Supplementary Tables

Supplementary Table S1. The list of antibodies used in the study

Antigen	Host	Company	Cat#/code
EZH2	Rabbit	Cell Signaling	5246
H3	Rabbit	Abcam	Ab1791
H3K27Me3	Rabbit	Millpore	17-622
H3K27Me2	Rabbit	BeacomBio	BIO2014917
H3K9Me3	Rabbit	Millpore	07-442
H3K4Me3	Mouse	Millpore	05-1339
H3K4Me2	Mouse	Millpore	05-1249
Cyclin D	Mouse	Cell Signaling	2926
Cyclin E	Mouse	BD	551160
CDK1	Rabbit	Abcam	ab32384
CDK2	Rabbit	Cell Signaling	2546
CDK4	Mouse	Abcam	ab108357
P21	Rabbit	Abcam	ab10919
BAX	Rabbit	Cell Signaling	5023
BCL-2	Rabbit	Cell Signaling	2870S
Caspase 3	Rabbit	Cell Signaling	9662
Caspase 9	Mouse	Cell Signaling	9508
Cleaved-Caspase9	Rabbit	Cell Signaling	7237
Ki67	Rabbit	Abcam	ab16667
β-actin	Mouse	Zsgb-Bio	TA-09

Supplementary Table S2. The list of primers used in the study

**qRT-PCR Primers**	
Genes	AB no.
CDH1	HQP023466
CDKN1C	HQP000356
CDKN2A	HQP000369
RUNX3	HQP021393
**siRNA sequence**	
Gene	Target sequences
EZH2-siRNA1	GACUCUGAAUGCAGUUGCU
EZH2-siRNA2	GCAAATTCTCGGTGTCAAA
EZH2-siRNA3	AUCAGCUCGUCUGAACCUCUU

Supplementary Table S3. HMT Profiling of ZLD1039 with a panel of HMT enzymes

Enzyme	ZLD1039 IC_{50}(nM)	Fold-selectivity	Positive control	IC_{50}(μM)	Assay with enzyme complex	Substrate
EZH2 WT	5.6	1	SAH	21	EZH2/EED/SUZ12/RbAp48/AEBP2	H3[21-24]
EZH2 A677G	4.0	0.71	SAH	21	EZH2/EED/SUZ12/RbAp48/AEBP2	H3[21-24]
EZH2 Y641F	15	2.68	SAH	66	EZH2/EED/SUZ12/RbAp48/AEBP2	H3[21-24]
EZH2 Y641S	71	12.68	SAH	20	EZH2/EED/SUZ12/RbAp48/AEBP2	H3[21-24]
EZH2 Y641C	210	37.5	SAH	111	EZH2/EED/SUZ12/RbAp48/AEBP2	H3[21-24]
EZH1	81	14.46	SAH	79	EZH1/EED/SUZ12/RbAp48/AEBP2	H3[21-24]
MLL1	26,500	4732	Chaetocin	0.13	MLL1/WDR5/Ash2L/RbBP5	H3[1-21]
G9a	>100,000	>17,000	SAH	0.60	G9a	H3[1-21]
PRDM9	>100,000	>17,000	SAH	50	PRDM9	H3[1-21]
SETD7	>100,000	>17,000	SAH	1.96	SETD7	H3[1-21]
SYMD2	>100,000	>17,000	SAH	1	SYMD2	H3[1-21]
SUV39H1	>100,000	>17,000	Chaetocin	0.68	SUV39H1	H3[1-21]
SUV39H2	>100,000	>17,000	SAH	3	SUV39H2	H3[1-21]
SETD8	>100,000	>17,000	SAH	71	SETD8	H3[6-27]
DOT1L	>100,000	>17,000	SAH	0.28	DOT1L	Oligonucleosome
NSD3	>100,000	>17,000	Chaetocin	0.59	NSD3	Oligonucleosome

Supplementary Table S4. Pharmacokinetic parameters for ZLD1039 in Sprague−Dawley rats

Parameter	IV(2mg/kg)	PO (100mg/kg)
AUC_{0-t}(ug*h)	556.4	4546.1
AUC_{0-inf} (ug*h)	556.4	12994.2
$t_{1/2}$ (h)	0.488	4.369
T_{max}(h)		0.25
C_{max}(ug/L)		482.1
MRT (h)	0.704	3.249
CL (L/h/kg)	0.004	
F(%)		16.3

Note: PO= oral delivery, IV= intravenous, AUC= area under the curve (measurement of exposure), t1/2= half life, Tmax=the time required to reach the maximum plasma concentration, MRT: the mean residence time, CL=plasma clearance, Cmax= maximum plasma concentration, F: oral bioavailability.

Supplementary Table S5. Microarray data including up-regulated and down-regulated genes

GS	FC	T-1	T-2	T-3	CTL-1	CTL-2	CTL-3	*P* value
FAM111B	9.04	6.097	6.226	6.594	2.964	3.290	3.163	0.0002
LGR5	6.76	6.769	6.977	7.517	4.072	4.703	4.249	0.0008
FAM111B	6.69	5.596	6.372	6.363	3.113	3.614	3.462	0.0021
POLE2	6.45	8.112	8.045	8.387	5.842	5.170	5.408	0.0011
SPRR1B	6.35	8.138	8.081	8.214	5.198	5.468	5.722	0.0019
CCNE2	5.30	5.768	5.709	6.457	3.388	3.793	3.631	0.0034
E2F8	4.59	7.029	6.986	7.352	5.379	4.532	4.745	0.0053
MNS1	4.56	6.738	7.249	6.875	4.870	4.686	4.778	0.0022
FABP5	4.20	9.689	9.780	10.095	7.913	7.391	8.010	0.0016
CDKN1C	4.08	8.767	8.553	9.041	6.897	6.758	6.656	0.4445
CDCA7	4.05	10.541	10.562	10.804	8.809	8.140	8.818	0.0061
SKP2	4.03	7.985	7.608	8.613	6.113	6.012	6.224	0.0183
LMNB1	3.92	8.773	8.588	9.161	6.960	6.810	6.895	0.0050
DSCC1	3.90	8.535	8.755	8.775	6.485	6.940	6.723	0.0007
CDKN2A	3.88	5.055	5.845	5.950	3.684	4.019	3.354	0.0840
CDKN2A	3.88	9.221	9.702	9.180	7.538	7.444	7.303	0.1169
HIBCH	3.87	8.860	9.474	8.935	7.234	7.218	7.036	0.0057
RRM2	3.84	10.857	10.685	10.960	9.057	8.555	9.022	0.0019
DLGAP5	3.76	7.852	8.531	8.274	6.372	6.441	6.176	0.0050
MYB	3.76	7.279	7.585	7.810	5.744	5.673	5.567	0.0033
DSCC1	3.70	8.780	9.072	9.271	7.243	7.204	7.049	0.0021
ANKRD32	3.69	6.187	6.612	6.860	4.699	4.857	4.505	0.0035
NDC80	3.66	7.853	8.254	7.548	6.329	5.753	5.992	0.0025
CDH1	3.64	7.489	8.120	7.936	6.002	6.166	5.838	0.0878
LMNB1	3.56	7.759	7.825	8.085	5.738	6.122	6.285	0.0015
LMNB1	3.56	7.756	7.822	8.082	5.747	6.119	6.276	0.0013
GINS2	3.54	9.407	9.198	9.418	7.551	6.989	7.885	0.0140
SPC25	3.51	9.398	9.597	9.503	7.729	7.470	7.849	0.0007
ANKRD32	3.51	5.759	6.296	6.708	4.658	4.688	4.055	0.0080
KIF20A	3.48	9.442	9.492	9.510	7.643	7.801	7.593	0.0005
CDC7	3.41	6.652	7.076	6.850	5.237	5.143	4.900	0.0005
FAM102B	3.39	7.575	7.545	7.805	5.962	5.906	5.789	0.0002
ESCO2	3.38	7.763	8.229	8.085	6.106	6.538	6.158	0.0008
KIAA0101	3.36	11.034	11.004	10.932	9.125	9.119	9.462	0.0025
TMX1	3.33	6.946	7.462	7.416	5.606	5.489	5.569	0.0070

MCM10	3.32	7.123	7.451	7.573	5.759	5.678	5.539	0.0017
MCM10	3.32	7.443	7.417	7.503	5.782	5.861	5.504	0.0026
KIF15	3.31	6.120	6.448	6.378	4.819	4.653	4.257	0.0021
RUNX3	3.28	11.108	11.075	10.682	9.142	9.131	9.464	0.2853
CLSPN	3.27	5.609	5.924	6.615	4.297	4.361	4.542	0.0254
CLDN2	3.25	7.619	7.745	8.030	6.149	6.236	5.924	0.0005
RRM2	3.24	11.247	11.205	11.442	9.696	9.511	9.606	0.0001
GINS4	3.23	7.728	7.694	7.985	6.269	5.901	6.161	0.0003
DTL	3.21	8.791	8.954	9.090	7.235	7.266	7.307	0.0017
CA12	3.20	7.196	7.154	7.211	5.444	5.319	5.728	0.0044
UBE2T	3.19	11.554	11.512	11.367	9.824	9.713	9.881	0.0000
HMMR	3.18	7.176	7.898	7.234	5.815	5.553	6.009	0.0073
RUNX3	7.61	7.271	5.877	5.842	5.459	3.838	3.838	7.3107
UCHL5	3.17	7.119	7.596	7.655	5.940	5.580	5.882	0.0022
CLDN2	3.17	9.375	9.342	9.430	7.476	7.844	7.809	0.0035
DTL	3.15	9.050	8.940	9.420	7.433	7.460	7.593	0.0040
MCM4	3.14	9.230	9.005	9.692	7.808	7.445	7.784	0.0049
PBK	3.12	8.822	9.066	9.057	7.488	7.224	7.313	0.0001
NEK2	3.12	8.856	9.201	8.925	7.411	7.592	7.028	0.0022
ERCC6L	3.11	6.646	6.741	7.297	4.846	5.653	5.259	0.0064
FSBP /// RAD54B	3.10	6.879	7.160	7.290	5.569	5.512	5.368	0.0014
MCM10	3.10	7.935	8.042	8.513	6.694	6.581	6.372	0.0038
REG4	3.08	11.369	11.295	10.963	9.519	9.478	9.779	0.0007
KIAA0101	3.07	7.701	7.856	7.942	6.259	5.960	6.401	0.0014
HPDL	3.06	8.410	8.105	8.586	6.763	6.383	7.077	0.0039
E2F8	3.06	7.068	7.119	7.512	5.409	5.941	5.496	0.0019
TYMS	3.05	9.448	9.287	9.629	7.930	7.416	8.125	0.0072
FABP5	3.05	9.887	9.813	10.350	8.342	8.136	8.742	0.0028
SLC12A2	3.05	9.532	9.632	9.645	8.133	7.971	7.872	0.0004
BLM	3.04	7.296	7.854	7.727	6.257	6.140	5.651	0.0031
LMNB1	3.03	8.036	7.908	8.162	6.548	6.376	6.387	0.0001
KIAA0101	3.02	11.318	11.337	11.289	9.702	9.488	9.936	0.0060
RBM45	3.00	7.733	8.004	7.870	6.299	6.631	5.813	0.0138
GINS1	3.00	6.371	6.127	7.158	4.907	4.915	5.262	0.0274
MCM2	2.95	8.988	8.720	8.978	7.452	7.053	7.472	0.0013
CA12	2.95	7.209	7.082	7.160	5.515	5.417	5.809	0.0030
MIS18BP1	2.94	6.019	6.774	6.413	4.958	4.688	4.973	0.0103
GINS4	2.93	5.991	6.270	6.155	4.738	4.288	4.703	0.0021
HELLS	2.92	8.284	8.721	8.499	7.213	6.768	6.882	0.0011
MCM4	2.92	9.030	8.804	9.235	7.509	7.240	7.684	0.0010

CDCA7	2.91	7.916	7.964	8.247	6.698	6.210	6.576	0.0016
DLEU2	2.89	7.434	7.836	8.024	6.333	6.215	6.209	0.0099
MCM4	2.89	9.960	9.729	10.336	8.556	8.396	8.542	0.0091
GINS4	2.88	5.850	6.034	6.016	4.548	4.241	4.520	0.0006
CDKN2AIPNL	2.88	12.280	12.141	12.501	10.779	10.727	10.858	0.2411
E2F8	2.88	6.672	6.689	6.980	5.270	5.170	5.340	0.0010
SESTD1	2.87	6.793	6.940	7.052	5.596	5.385	5.226	0.0006
KIAA0101	2.83	12.611	12.539	12.622	11.138	10.805	11.280	0.0070
HMMR	2.83	9.168	9.778	9.316	7.967	8.046	7.801	0.0079
MCM6	2.83	9.838	9.925	9.952	8.602	8.262	8.327	0.0023
GINS1	2.83	9.546	9.435	10.056	8.175	8.144	8.292	0.0128
CCNA2	2.83	9.312	9.404	9.436	7.887	7.954	7.812	0.0000
HELLS	2.82	6.625	7.018	6.617	5.379	5.322	5.093	0.0014
KIAA1199	2.82	7.479	7.630	7.522	6.156	5.924	6.066	0.0001
MCM3	2.81	10.367	10.226	10.429	8.970	8.603	8.962	0.0018
RIBC2	2.79	7.138	6.971	7.456	5.477	5.623	6.008	0.0023
SLC40A1	2.78	7.140	7.529	7.190	5.702	5.929	5.831	0.0016
MCM6	2.77	8.128	8.083	8.304	6.937	6.453	6.680	0.0029
RUNX3	2.76	9.611	9.885	9.875	8.303	8.378	8.308	9.6111
RMI1	2.76	8.090	8.450	8.231	6.749	6.849	6.800	0.0031
MKI67	2.75	6.390	6.694	6.896	5.354	5.090	5.190	0.0031
SMC2	2.73	6.110	7.069	6.508	5.135	5.476	4.823	0.0176
HAUS7	2.72	8.057	7.914	8.232	6.574	6.438	6.841	0.0008
RFC3	2.72	9.480	9.497	9.606	8.016	8.099	8.144	0.0000
MCM4	2.71	9.835	9.584	10.027	8.392	8.142	8.595	0.0014
MSH2	2.70	7.758	8.254	8.130	6.732	6.788	6.326	0.0023
HSPH1	2.70	9.781	10.025	10.464	8.557	9.000	8.438	0.0060
RAB27B	2.69	5.555	5.703	5.870	3.997	4.080	4.679	0.0113
DNA2	2.69	6.355	6.615	6.784	5.122	5.314	5.053	0.0015
HSPH1	2.69	7.619	8.065	8.709	6.825	6.993	6.453	0.0312
TMEM14A	2.68	11.083	10.985	11.528	9.754	9.710	9.913	0.0075
IL20RA	2.68	6.935	7.261	6.968	5.560	5.817	5.532	0.0005
NEK2	2.67	8.770	9.094	8.708	7.489	7.654	7.169	0.0018
CDC25A	2.66	6.899	6.790	7.548	5.649	5.493	5.941	0.0130
ARHGAP11A	2.66	6.491	6.949	6.782	5.167	5.567	5.256	0.0015
MKI67	2.66	8.153	8.195	8.373	6.782	6.871	6.847	0.0007
DNA2	2.66	6.871	7.165	7.218	5.734	5.714	5.597	0.0023
RFC4	2.66	10.219	10.372	10.344	9.021	8.902	8.780	0.0002
SLC12A2	2.65	7.391	8.196	7.501	6.544	6.191	6.242	0.0193

MCM2	2.65	8.963	8.726	8.932	7.477	7.054	7.788	0.0135
HSPH1	2.64	8.731	8.963	9.554	7.759	7.984	7.360	0.0128
UCHL5	2.64	7.209	7.559	7.253	5.791	6.217	5.802	0.0018
NCAPH	2.63	9.543	9.391	9.536	8.001	8.176	8.103	0.0000
UCHL5	2.63	9.066	9.532	9.224	7.928	8.056	7.661	0.0017
SPC24	2.63	8.795	8.782	8.895	7.441	7.017	7.743	0.0188
FAS	2.62	6.793	7.098	7.155	5.904	5.469	5.494	0.0018
UCHL5	2.61	6.810	7.038	7.142	5.767	5.616	5.457	0.0005
TIPIN	2.60	8.587	8.756	8.994	7.329	7.451	7.443	0.0039
OAS3	2.60	7.943	7.917	8.052	6.623	6.426	6.717	0.0009
MCM7	2.60	10.729	10.573	10.925	9.393	9.140	9.552	0.0010
TMX1	2.60	9.313	9.621	9.418	8.060	8.309	7.834	0.0020
TYMS	2.59	10.768	10.656	10.935	9.534	9.112	9.560	0.0031
HMMR	2.59	7.030	7.801	7.350	5.873	6.156	6.127	0.0161
MSH2	2.58	7.914	8.397	8.232	6.902	7.016	6.516	0.0027
CCNA2	2.58	9.588	9.679	9.690	8.247	8.418	8.181	0.0006
DEPDC7	2.58	6.141	6.807	6.846	5.368	5.519	4.821	0.0121
MCM4	2.58	10.819	10.590	11.042	9.567	9.205	9.587	0.0016
DNMT1	2.57	8.399	8.186	8.295	7.047	6.943	6.794	0.0002
CHRNA5	2.57	7.646	7.957	8.003	6.408	6.435	6.688	0.0009
SPAG5	2.57	9.356	9.428	9.428	8.161	7.935	8.031	0.0008
KIAA0101	2.56	11.904	12.249	12.217	10.675	10.616	10.999	0.0011
ATAD2	2.56	8.121	8.647	8.388	7.044	7.261	6.786	0.0028
DUT	2.56	8.512	8.423	8.651	7.190	6.944	7.359	0.0019
HSP90AA1	2.56	11.077	11.442	11.404	9.976	10.129	9.753	0.0011
MSH6	2.56	9.400	9.565	9.791	8.271	8.395	8.024	0.0010
ORC1	2.55	8.392	8.244	8.717	7.059	7.015	7.256	0.0033
ASPM	2.55	6.195	6.863	6.662	5.032	5.307	5.388	0.0089
ATAD2	2.55	9.173	9.732	9.460	8.009	8.400	7.917	0.0036
RAD51AP1	2.55	8.262	8.692	8.443	7.204	6.917	7.243	0.0013
TMEM201	2.54	7.534	7.082	7.766	6.022	6.076	6.308	0.0117
HOXA13	2.54	8.816	8.930	9.143	7.661	7.731	7.464	0.0005
UCHL5	2.53	8.184	8.714	8.473	7.152	7.346	6.854	0.0031
HSPH1	2.53	8.370	8.678	9.294	7.324	7.768	7.342	0.0234
WDR67	2.53	5.835	6.320	5.803	4.770	4.637	4.586	0.0098
GPSM2	2.53	9.027	9.368	9.329	8.066	8.045	7.571	0.0037
HAT1	2.52	9.569	10.155	10.008	8.506	8.772	8.492	0.0069
SHCBP1	2.52	7.645	7.902	7.997	6.666	6.550	6.323	0.0008
KIF23	2.52	7.627	8.355	7.991	6.853	7.012	6.018	0.0269
NEK2	2.52	8.375	8.679	8.287	7.198	7.301	6.833	0.0022

SGK494	2.52	8.053	8.035	8.328	6.793	6.756	6.888	0.0018
SMC3	2.52	6.126	7.126	6.515	5.228	5.639	5.014	0.0267
RAD51AP1	2.51	8.264	8.662	8.463	7.215	6.936	7.255	0.0010
BRI3BP	2.51	7.806	7.758	7.905	6.565	6.205	6.674	0.0068
PRIM2	2.51	7.983	7.893	8.176	6.829	6.417	6.797	0.0021
DBF4	2.51	7.487	8.259	7.835	6.492	6.932	6.175	0.0133
HSPH1	2.51	8.979	9.268	9.809	7.948	8.377	7.813	0.0150
MAD2L1	2.51	10.201	10.389	10.451	8.999	9.079	8.992	0.0012
MSH2	2.51	7.926	8.233	8.009	6.596	7.055	6.496	0.0060
HAUS7	2.51	8.386	8.248	8.644	7.129	6.848	7.316	0.0019
TUBB	2.50	11.817	11.618	12.017	10.400	10.527	10.576	0.0025
MAD2L1	2.50	9.710	9.889	9.981	8.517	8.460	8.646	0.0003
BMP4	2.49	11.504	11.364	11.761	10.207	10.252	10.251	0.0070
NUSAP1	2.49	9.651	9.805	9.797	8.416	8.488	8.411	0.0002
HAUS7	2.49	8.795	8.717	8.949	7.583	7.217	7.687	0.0042
SMC3	2.49	7.601	8.451	7.843	6.515	6.986	6.540	0.0186
MORC4	2.48	10.310	10.540	10.415	9.129	9.185	9.019	0.0002
FAM83D	2.48	9.784	9.667	10.241	8.722	8.530	8.567	0.0113
CDKN2A	2.48	8.324	9.222	8.494	7.465	7.794	6.873	0.0447
SLC12A2	2.48	9.693	10.284	9.779	8.649	8.857	8.351	0.0061
HAT1	2.47	9.470	10.070	9.837	8.459	8.644	8.404	0.0092
RMI1	2.47	6.703	6.909	7.130	5.975	5.461	5.340	0.0075
IL20RA	2.47	7.273	7.335	7.291	5.872	6.032	6.072	0.0010
SPRR3	2.47	6.420	6.055	6.405	4.945	4.856	5.177	0.0013
NEK2	2.47	8.972	9.406	8.994	7.844	7.923	7.731	0.0057
HMGN3	2.47	7.588	7.838	7.836	6.678	6.397	6.261	0.0016
CEP55	2.45	8.371	8.713	8.576	7.319	7.534	6.882	0.0090
TMEM48	2.44	9.863	10.079	9.994	8.715	8.776	8.581	0.0001
SASS6	2.43	6.485	7.223	6.760	5.431	5.862	5.369	0.0111
SPRR1A	2.43	7.347	7.101	7.371	6.001	5.964	6.023	0.0034
MLF1IP	2.42	6.760	7.043	6.565	5.757	5.354	5.435	0.0025
BRIX1	2.42	10.246	10.534	10.509	9.099	9.408	8.933	0.0027
OPA1	2.42	6.929	7.575	7.082	6.025	5.780	6.023	0.0131
ZNF367	2.42	6.656	6.724	7.482	5.948	5.834	5.346	0.0224
ZWILCH	2.42	8.118	8.659	8.458	7.261	7.151	7.050	0.0083
LRP8	2.41	9.719	9.534	9.804	8.360	8.225	8.639	0.0018
MCM2	2.41	9.938	9.652	9.772	8.525	8.261	8.737	0.0030
BUB1B	2.41	9.257	9.331	9.638	8.185	8.317	7.910	0.0016
NIN	2.41	7.377	7.991	7.560	6.238	6.686	6.215	0.0066
CDKN2AIP	2.41	7.201	7.099	6.805	5.560	5.892	5.853	0.6973
NEK2	2.40	8.354	8.712	8.423	7.066	7.555	7.047	0.0050
RMI1	2.40	5.760	6.506	6.318	5.346	4.836	4.623	0.0154

MCM8	2.39	7.235	7.778	7.708	6.504	6.374	6.094	0.0054
TGFBR3	2.39	6.992	6.852	7.227	5.701	5.867	5.756	0.0026
ZNF615	2.38	4.805	5.681	4.884	3.855	3.873	4.043	0.0449
SERPIND 1	2.38	6.803	6.723	6.762	5.578	5.353	5.591	0.0020
SLC35G1	2.38	7.576	7.707	8.301	6.818	6.649	6.456	0.0180
SKP2	2.38	6.615	6.663	6.895	5.606	5.335	5.491	0.0005
MORC4	2.37	9.904	10.192	9.964	8.941	8.826	8.538	0.0016
TOPBP1	2.37	8.081	8.562	8.416	7.062	7.370	6.893	0.0034
DLEU2	2.37	7.004	7.584	7.687	6.411	5.948	6.236	0.0124
OAS1	2.37	8.510	8.609	8.433	7.369	7.110	7.333	0.0005
CEP55	2.36	8.820	9.239	9.161	7.707	8.196	7.547	0.0087
MKI67	2.36	8.329	8.718	8.486	7.394	7.433	6.965	0.0034
PDE3B	2.36	7.335	7.218	7.730	6.167	6.495	5.885	0.0063
ZNF614	2.36	7.705	8.119	7.977	6.574	6.878	6.639	0.0017
CDCA2	2.36	5.501	5.641	5.799	4.474	4.435	4.327	0.0011
SFXN1	2.36	9.733	9.697	9.999	8.595	8.575	8.562	0.0056
SHCBP1	2.36	7.170	7.255	7.589	6.226	6.294	5.763	0.0050
CYP1B1	2.36	7.535	8.013	7.608	6.879	6.056	6.441	0.0161
CDKN2A IPNL	2.36	10.219	9.865	10.275	8.976	8.503	9.132	0.0821
HSPH1	2.36	9.961	10.284	10.687	9.034	9.386	8.842	0.0115
KIAA152 4	2.35	5.038	5.838	5.526	4.334	4.545	3.842	0.0176
ZWILCH	2.35	7.873	8.190	8.061	6.784	6.943	6.701	0.0006
MORC4	2.35	9.864	10.083	9.883	8.778	8.757	8.597	0.0002
UHRF1	2.35	8.585	8.303	8.851	7.342	7.247	7.492	0.0076
SLBP	2.35	9.450	9.327	9.854	8.424	8.368	8.186	0.0077
GCFC1	2.35	6.463	7.007	7.148	5.516	5.861	5.611	0.0148
KNTC1	2.35	6.777	7.446	7.072	5.978	5.938	5.755	0.0161
MORC4	2.35	10.555	10.868	10.779	9.509	9.517	9.502	0.0057
SSX2IP	2.35	6.626	7.151	6.935	5.647	5.843	5.563	0.0054
UCHL5	2.35	7.031	7.368	7.253	6.127	6.081	5.744	0.0016
MELK	2.34	8.428	8.601	8.406	7.362	7.321	7.058	0.0009
CKAP2	2.34	7.047	7.942	7.700	6.333	6.711	6.033	0.0256
ZWINT	2.34	9.113	9.162	9.164	8.014	7.668	8.055	0.0088
HSPA8 D	2.33	5.448	5.580	5.920	4.547	4.606	4.124	0.0042
HSP90A A4P	2.33	11.241	11.689	11.534	10.198	10.579	9.996	0.0056
SFXN1	2.33	8.782	8.737	8.714	7.475	7.671	7.410	0.0026
ZWINT	2.33	11.914	11.866	12.134	10.934	10.420	10.862	0.0065
MIS18BP 1	2.33	4.748	5.535	5.306	4.085	3.733	4.187	0.0185
CDC25A	2.33	8.331	7.963	8.931	7.267	7.031	7.415	0.0392

MCM7	2.33	10.905	10.837	10.972	9.648	9.430	9.941	0.0102
TOP2A	2.33	9.480	10.089	9.720	8.706	8.784	8.130	0.0114
SPRR1A	2.32	5.978	5.575	5.936	4.966	4.625	4.177	0.0161
CNN3	2.32	9.712	9.827	9.829	8.635	8.637	8.445	0.0003
CCNB1	2.32	11.951	11.933	11.989	10.731	10.789	10.707	0.0000
FAS	2.32	7.174	7.442	7.411	6.384	6.181	5.767	0.0103
UHRF1	2.32	10.280	9.930	10.573	9.226	8.820	9.137	0.0086
MIR17HG	2.32	5.739	6.210	6.116	4.940	5.068	4.364	0.0127
NEK2	2.32	9.912	10.311	9.970	8.862	9.092	8.591	0.0034
TOP2A	2.32	8.889	9.471	9.044	8.064	8.028	7.718	0.0072
BARD1	2.31	8.199	8.385	8.199	7.108	7.256	6.751	0.0072
MCM3	2.31	10.551	10.209	10.509	9.306	8.934	9.383	0.0029
DBF4	2.31	6.944	7.528	7.218	6.053	6.168	5.887	0.0088
TFRC	2.31	8.883	9.263	9.163	7.958	8.128	7.568	0.0055
SLBP	2.31	10.256	10.124	10.703	9.122	9.341	9.046	0.0093
CYP1B1	2.31	8.734	9.240	8.876	7.860	7.473	7.905	0.0042
RFC3	2.31	10.197	10.345	10.320	9.075	9.146	9.024	0.0001
MCM3	2.31	11.156	10.972	11.241	10.106	9.561	10.042	0.0091
CENPF	2.30	6.982	7.636	7.202	5.967	6.324	5.964	0.0102
MSH6	2.30	7.817	8.102	8.381	6.963	7.060	6.695	0.0056
TBC1D4	2.30	8.566	8.665	8.907	7.687	7.673	7.119	0.0098
HMMR	2.30	7.652	8.277	7.804	6.709	6.779	6.712	0.0233
METTL7B	2.30	8.746	8.560	8.795	7.315	7.389	7.760	0.0043
MRPL17	2.30	11.628	11.536	11.652	10.423	10.270	10.508	0.0006
MORC4	2.30	10.856	11.097	11.042	9.928	9.880	9.572	0.0016
DBF4	2.30	7.617	8.260	8.043	6.578	7.286	6.357	0.0273
EXO1	2.30	7.586	7.694	7.735	6.400	6.486	6.531	0.0000
MCM8	2.30	6.938	7.352	7.278	6.205	6.177	5.525	0.0153
UNG	2.30	10.342	10.074	10.550	9.267	8.754	9.322	0.0073
ANLN	2.29	9.511	10.020	10.041	8.529	9.007	8.435	0.0084
SMARCE1	2.29	8.165	8.494	8.323	7.028	7.321	7.050	0.0009
SPC24	2.29	7.610	7.609	7.902	6.730	6.373	6.432	0.0014
HSP90AA1	2.28	8.033	8.454	8.359	7.005	7.275	7.010	0.0025
BUB1	2.28	8.762	8.938	9.071	7.769	7.884	7.546	0.0009
CD47	2.28	6.985	6.973	7.274	6.056	5.960	5.641	0.0021
OAS3	2.28	9.556	9.413	9.537	8.436	7.991	8.474	0.0113
DBF4	2.28	6.591	7.257	7.041	5.606	6.271	5.372	0.0256
CDH1	2.28	7.489	8.156	7.566	6.595	6.829	6.270	0.3025
MAD2L1	2.28	10.703	10.836	10.875	9.559	9.702	9.596	0.0001
CENPH	2.28	9.556	9.588	9.566	8.411	8.241	8.488	0.0033

NUDT6	2.28	6.239	6.363	6.589	5.454	4.960	5.199	0.0035
TOP2A	2.27	9.407	9.991	9.459	8.477	8.570	8.319	0.0150
BRI3BP	2.27	8.067	7.912	8.471	7.049	6.570	7.264	0.0119
BRI3BP	2.27	9.129	9.029	9.314	8.055	7.774	8.097	0.0010
TUBGCP5	2.26	6.424	6.483	6.332	5.451	5.224	4.995	0.0070
HMGN3	2.26	10.086	10.190	10.312	9.068	9.052	8.939	0.0003
DEPDC7	2.26	6.148	6.509	6.871	5.247	5.659	5.121	0.0132
CDCA3	2.26	8.668	8.748	9.038	7.602	7.480	7.841	0.0016
SMC4	2.26	7.362	8.265	7.560	6.748	6.788	6.228	0.0320
PCYOX1	2.25	7.998	7.935	8.200	7.052	6.910	6.634	0.0024
SMC3	2.25	6.568	7.462	6.682	5.773	6.115	5.392	0.0346
HSPH1	2.25	10.043	10.211	10.733	9.114	9.487	8.905	0.0135
CKAP2	2.25	8.288	8.904	8.766	7.434	7.799	7.229	0.0099
PARP9	2.25	6.949	7.002	7.061	6.152	5.866	5.390	0.0304
CDKN3	2.25	7.832	7.993	8.259	6.815	6.838	6.957	0.0062
RAPGEF5	2.25	7.453	7.589	7.379	6.222	6.715	5.863	0.0330
MIS18A	2.25	8.187	8.156	8.396	7.169	7.082	6.994	0.0005
REG4	2.24	5.826	5.047	5.512	4.311	4.516	4.142	0.0221
WDR76	2.24	7.744	7.495	7.863	6.792	6.240	6.549	0.0056
TTK	2.24	7.864	8.457	8.111	6.905	7.235	6.838	0.0074
BRIX1	2.24	10.736	11.019	10.910	9.709	10.027	9.393	0.0121
CKAP2	2.23	8.319	8.766	8.734	7.410	7.740	7.179	0.0061
C2orf18	2.23	10.448	10.421	10.503	9.364	9.207	9.325	0.0002
FAM217B	2.23	6.640	6.890	7.325	5.717	6.127	5.571	0.0126
C15orf23	2.23	10.200	10.304	10.224	9.016	9.295	8.930	0.0056
KIF23	2.23	8.189	8.873	8.628	7.484	7.665	7.102	0.0126
SEPT11	2.23	8.405	8.726	8.666	7.578	7.488	7.270	0.0010
SEPT11	2.23	7.342	7.534	7.381	6.086	6.550	6.117	0.0086
C3orf58	2.23	5.990	6.238	6.500	5.262	5.283	4.691	0.0104
FEN1	2.22	10.375	10.012	10.660	9.176	9.242	9.243	0.0252
DIXDC1	2.22	4.510	4.434	5.157	3.736	3.654	3.340	0.0222
KLHL23	2.22	8.244	8.361	8.746	7.288	7.235	7.416	0.0104
GLMN	2.22	6.774	7.109	7.079	5.756	5.936	5.838	0.0028
RAD51AP1	2.22	8.056	8.452	8.283	7.203	6.889	7.250	0.0021
CKAP2	2.22	6.723	7.349	7.026	5.875	6.190	5.597	0.0101
SGOL2	2.22	4.396	5.208	5.083	3.599	3.926	3.825	0.0347
DBF4	2.21	7.560	8.141	7.746	6.682	6.908	6.442	0.0074
VRK1	2.21	7.130	7.784	7.462	6.222	6.379	6.402	0.0199
DEPDC1B	2.21	9.313	9.584	9.455	8.254	8.390	8.280	0.0009

ASF1B	2.21	8.607	8.466	8.844	7.563	6.983	7.838	0.0280
KIF11	2.21	8.434	8.802	8.847	7.585	7.662	7.434	0.0047
TIPIN	2.21	8.325	8.252	8.484	7.234	7.362	7.028	0.0011
SEMA3A	2.21	7.048	7.663	7.135	6.332	6.216	5.921	0.0118
KRR1	2.21	7.657	8.544	7.906	6.971	7.046	6.806	0.0455
PMS1	2.20	6.628	7.310	7.044	5.825	6.101	5.682	0.0133
HMGCR	2.20	8.177	8.462	8.235	7.100	7.366	6.974	0.0019
HSPA4L	2.20	7.282	7.851	7.835	6.555	6.819	6.172	0.0126
GEMIN5	2.20	9.048	9.029	9.543	8.163	8.083	8.014	0.0170
HMGCR	2.20	8.583	8.799	8.447	7.304	7.857	7.183	0.0162
HNMT	2.20	5.045	5.538	4.751	4.000	4.063	3.972	0.0393
KIAA1586	2.20	5.471	6.254	6.228	4.842	4.953	4.876	0.0489
TMPO	2.20	7.789	7.997	8.135	6.921	6.823	6.791	0.0035
ANLN	2.20	7.981	8.521	8.526	7.007	7.538	7.087	0.0101
FANCI	2.19	8.657	8.918	8.795	7.832	7.766	7.340	0.0077
HAUS7	2.19	8.469	8.355	8.660	7.487	7.213	7.393	0.0007
MCM7	2.19	12.626	12.394	12.629	11.481	11.243	11.529	0.0007
GCA	2.19	7.436	7.924	7.466	6.398	6.696	6.370	0.0063
KBTBD6	2.19	6.275	6.717	6.601	5.306	5.580	5.337	0.0036
CENPQ	2.18	6.777	7.246	7.025	5.476	6.395	5.662	0.0352
NUSAP1	2.18	8.934	8.979	8.945	7.870	7.929	7.675	0.0037
MIS18BP1	2.18	4.507	5.315	4.932	3.799	3.940	3.748	0.0365
LRP8	2.18	8.822	8.366	8.668	7.430	7.271	7.772	0.0050
NPNT	2.18	8.163	8.344	8.224	7.220	7.146	6.990	0.0003
C2orf18	2.18	10.090	10.237	10.091	9.110	9.020	8.920	0.0001
NUDT6	2.18	6.503	6.503	6.824	5.807	5.182	5.430	0.0103
NUSAP1	2.18	9.406	9.630	9.559	8.454	8.427	8.356	0.0009
ZNF30	2.18	5.306	5.887	5.323	4.381	4.665	4.135	0.0116
SMC4	2.17	7.697	8.341	8.165	7.102	7.052	6.736	0.0131
MCM8	2.17	6.193	6.739	6.532	5.662	5.311	5.131	0.0073
LOC728802	2.17	5.662	5.922	5.783	4.897	4.469	4.626	0.0033
LEO1	2.17	6.824	7.472	6.843	5.990	5.955	5.940	0.0359
EED	2.17	9.148	9.552	9.419	8.279	8.293	8.221	0.0094
HSPH1	2.17	10.037	10.183	10.641	9.195	9.453	8.875	0.0110
C9orf100	2.17	8.106	8.091	7.947	7.146	6.437	7.112	0.0330
CDCA2	2.17	7.854	8.143	8.166	6.997	7.156	6.634	0.0059
MCM7	2.17	12.139	11.945	12.136	10.985	10.710	11.152	0.0047
ATG4C	2.17	5.861	6.357	5.843	5.207	4.863	4.652	0.0089
OSR2	2.16	6.150	6.033	6.005	4.510	5.117	5.139	0.0266
LOC28554	2.16	6.172	6.928	6.452	5.027	5.559	5.652	0.0205

FAM98B	2.16	9.159	9.506	9.470	8.393	8.365	8.034	0.0022
BCCIP	2.16	6.663	6.910	7.130	5.615	6.230	5.431	0.0237
KRR1	2.16	7.343	8.016	7.616	6.634	6.878	6.096	0.0217
SYNCRIP	2.16	9.403	9.672	9.688	8.377	8.693	8.347	0.0017
FEN1	2.16	11.143	10.958	11.533	10.258	10.072	10.017	0.0126
C1orf112	2.16	7.906	7.914	8.143	6.921	6.903	6.814	0.0016
SNRNP25	2.16	10.505	10.442	10.685	9.510	9.106	9.642	0.0098
LOC100652751	2.16	8.785	8.862	8.489	7.617	7.480	7.726	0.0025
CKAP2	2.16	7.866	8.307	8.135	6.938	7.292	6.726	0.0070
C11orf82	2.16	7.519	7.655	7.774	6.550	6.647	6.429	0.0004
FANCL	2.15	7.773	8.147	7.938	6.852	6.755	6.955	0.0028
KIF14	2.15	5.581	6.200	5.969	5.091	4.613	4.757	0.0101
VSNL1	2.15	5.957	6.191	6.365	5.089	4.961	5.172	0.0037
BRCC3	2.15	6.163	6.506	6.465	5.254	5.501	5.062	0.0029
SLC40A1	2.15	7.518	7.897	7.455	6.350	6.776	6.444	0.0044
BRIX1	2.14	8.519	8.780	8.762	7.441	7.891	7.386	0.0084
ACN9	2.14	7.952	8.024	7.845	6.966	6.724	6.826	0.0004
ANLN	2.14	8.852	9.504	9.442	8.061	8.545	7.903	0.0182
CDC7	2.14	4.349	4.799	4.555	3.548	3.561	3.325	0.0042
HSP90AA4P	2.14	9.311	9.768	9.505	8.279	8.756	8.237	0.0075
TMEM48	2.14	9.516	9.755	9.723	8.520	8.646	8.548	0.0010
BCAS2	2.13	8.986	9.468	9.067	8.097	8.370	7.755	0.0098
C2orf18	2.13	10.330	10.477	10.353	9.324	9.315	9.243	0.0002
TRIM34	2.13	7.334	7.343	7.532	6.379	6.201	6.355	0.0002
RECQL	2.13	7.882	8.706	8.181	7.138	7.548	6.848	0.0279
CENPK	2.13	8.346	8.706	8.647	7.472	7.288	7.670	0.0022
ZWILCH	2.13	7.788	8.083	8.078	6.812	6.969	6.915	0.0026
SMC2	2.13	7.304	7.974	7.505	6.413	6.773	6.382	0.0152
MIS18A	2.12	7.950	8.079	8.143	6.931	7.044	6.942	0.0003
CROT	2.12	5.014	5.521	5.556	4.131	4.408	4.344	0.0132
FANCI	2.12	7.774	7.796	7.868	6.733	6.827	6.618	0.0007
PDE3B	2.12	6.305	6.530	7.022	5.581	5.369	5.729	0.0218
TMPO	2.12	6.872	6.763	7.319	6.094	5.871	5.785	0.0109
TUBB	2.12	12.259	12.120	12.492	11.068	11.264	11.305	0.0021
SYNCRIP	2.12	7.607	7.908	7.656	6.797	6.780	6.316	0.0075
MAP3K5	2.12	6.264	6.604	6.351	5.119	5.658	5.155	0.0100
HPSE	2.11	6.788	6.622	6.944	5.605	5.706	5.812	0.0013
FEN1	2.11	11.016	10.762	11.338	9.927	9.972	10.032	0.0209
SPC24	2.11	7.452	7.014	7.330	6.332	5.644	6.484	0.0320

SGK1	2.11	8.225	8.148	8.503	7.258	7.151	7.250	0.0059
ANKRD32	2.11	6.647	7.265	7.180	6.002	5.950	5.975	0.0314
XRCC4	2.11	5.605	6.291	6.049	4.948	4.828	5.007	0.0280
SLC5A3	2.11	7.609	8.301	8.146	7.028	7.065	6.803	0.0252
FAM64A	2.11	9.801	9.627	9.890	8.715	8.493	8.867	0.0019
CDKN2C	2.11	7.620	7.584	7.729	6.588	6.306	6.776	0.0099
C9orf41	2.11	7.182	7.507	7.815	6.242	6.712	6.348	0.0115
ERVMER34-1	2.10	7.460	7.268	7.998	6.618	6.627	6.345	0.0270
IFI30	2.10	9.952	9.924	10.115	8.861	8.717	9.163	0.0063
NASP	2.10	11.678	11.671	11.842	10.751	10.717	10.501	0.0006
MMS22L	2.10	6.690	7.078	6.898	6.000	5.819	5.635	0.0023
HMGCR	2.10	9.196	9.415	9.102	8.167	8.409	7.897	0.0061
CSE1L	2.10	11.200	11.583	11.370	10.413	10.459	10.063	0.0032
TMPO	2.10	9.600	9.729	9.820	8.765	8.512	8.658	0.0004
LTV1	2.10	6.465	6.902	6.893	5.792	5.817	5.456	0.0052
UBR7	2.10	7.831	7.861	8.026	6.813	6.703	6.989	0.0008
CYP1B1	2.10	8.735	9.143	8.892	7.987	7.741	7.854	0.0034
SCD	2.10	9.130	9.105	8.923	7.960	7.770	8.199	0.0045
SF3A3	2.10	9.919	9.876	9.972	8.834	8.871	8.861	0.0001
SETMAR	2.09	6.254	6.427	6.486	5.353	5.610	4.935	0.0213
CHEK1	2.09	9.194	9.399	9.318	8.294	8.350	8.058	0.0011
CACYBP	2.09	9.109	9.088	9.380	8.099	8.158	8.143	0.0063
RPAP3	2.09	7.075	7.648	7.233	6.323	6.403	6.079	0.0111
PCDHB16	2.09	5.837	6.066	6.154	5.274	4.644	4.895	0.0135
TMEM48	2.09	9.979	10.218	10.292	9.040	9.170	9.103	0.0034
WDR67	2.09	6.721	7.152	6.797	5.920	5.915	5.674	0.0048
PANK1	2.09	7.839	7.904	8.339	7.077	6.972	6.898	0.0145
CHEK1	2.09	8.906	8.969	9.070	7.978	7.973	7.811	0.0001
G2E3	2.08	5.552	6.264	6.184	4.785	5.215	4.885	0.0249
ZWILCH	2.08	9.190	9.470	9.370	8.261	8.487	8.091	0.0024
LGR4	2.08	7.926	8.157	8.234	7.078	7.333	6.672	0.0166
FAM69A	2.08	5.157	5.693	5.960	4.353	4.921	4.403	0.0274
WDHD1	2.08	4.914	5.888	5.382	4.485	4.619	3.999	0.0459
FASTKD3	2.08	8.601	8.861	8.658	7.655	7.863	7.405	0.0047
C1orf112	2.08	7.359	7.347	7.415	6.058	6.485	6.372	0.0122
HMGB1	2.08	8.410	8.913	8.656	7.702	7.776	7.336	0.0062
SGK1	2.08	8.380	8.488	8.840	7.472	7.446	7.650	0.0078
DEPDC1	2.08	6.698	7.258	6.899	6.064	6.035	5.596	0.0093
CCDC77	2.08	5.151	5.753	5.633	4.458	4.554	4.423	0.0260
CYP1B1	2.08	9.424	9.757	9.304	8.556	8.231	8.549	0.0044

CCDC109B	2.08	9.685	9.735	9.635	8.579	8.356	8.904	0.0187
SVIP	2.08	6.857	7.407	7.375	6.143	6.317	6.071	0.0171
SLC39A10	2.08	7.676	8.038	7.738	6.802	6.837	6.675	0.0047
CD24	2.08	10.262	10.344	10.479	9.320	9.413	9.193	0.0003
CNP	2.07	11.307	11.137	11.427	10.293	10.035	10.378	0.0016
NEIL3	2.07	4.305	4.417	4.249	3.282	3.413	3.111	0.0015
ASF1B	2.07	9.140	8.855	9.392	8.182	7.627	8.374	0.0215
TSPAN12	2.07	6.387	7.129	6.763	5.700	5.984	5.494	0.0209
CDC7	2.07	5.529	5.754	5.724	4.883	4.461	4.481	0.0064
SEPT11	2.07	7.326	7.613	7.537	6.226	6.840	6.174	0.0246
IL17RB	2.07	7.512	7.594	7.691	6.718	6.249	6.644	0.0111
COQ3	2.07	9.082	9.164	9.142	8.257	7.781	8.162	0.0161
WDR76	2.07	7.670	7.453	7.745	6.647	6.281	6.769	0.0066
VANGL1	2.07	8.843	8.649	9.126	7.845	7.852	7.820	0.0171
C4orf27	2.07	8.339	8.669	8.357	7.633	7.319	7.269	0.0026
HSPA4L	2.06	6.321	7.030	6.628	5.657	5.721	5.544	0.0319
CCDC77	2.06	6.682	6.895	6.722	6.075	5.742	5.242	0.0389
SQLE	2.06	10.519	10.372	10.538	9.444	9.436	9.428	0.0024
WDR75	2.06	7.742	8.415	7.604	6.784	7.248	6.673	0.0344
CALM1	2.06	10.180	10.368	10.435	9.322	9.330	9.216	0.0014
DBF4	2.06	8.198	8.809	8.583	7.450	7.766	7.270	0.0119
PARP1	2.06	10.600	10.276	10.690	9.566	9.366	9.535	0.0057
HAUS1	2.06	9.561	9.768	9.635	8.673	8.561	8.614	0.0006
DCK	2.05	8.362	8.528	8.609	7.467	7.528	7.396	0.0010
DHX9	2.05	8.321	8.688	8.797	7.813	7.626	7.237	0.0100
TSEN2	2.05	7.857	8.194	8.056	6.947	7.116	6.945	0.0021
SMC2	2.05	6.948	7.619	7.135	6.268	6.307	6.091	0.0271
TRMT10C	2.05	8.299	8.621	8.794	7.527	7.815	7.248	0.0092
DFFB	2.05	6.099	5.781	6.583	5.153	4.942	5.346	0.0317
SHPK	2.05	8.478	8.084	8.417	7.311	7.000	7.541	0.0074
RDM1	2.05	5.815	5.863	5.847	4.911	4.561	4.924	0.0118
SSX2IP	2.05	6.808	7.489	7.203	5.934	6.404	6.102	0.0168
DHX9	2.05	6.680	6.891	6.995	5.895	5.949	5.618	0.0018
CSE1L	2.05	9.953	10.476	10.147	9.036	9.444	9.003	0.0079
TFRC	2.05	11.939	12.180	11.983	10.969	11.258	10.743	0.0087
SYNCRIP	2.04	8.754	9.019	8.927	7.886	8.051	7.654	0.0029
CASP8AP2	2.04	5.991	6.170	6.123	5.324	4.606	5.171	0.0337
PPIP5K2	2.04	6.985	7.604	7.460	6.456	6.435	6.109	0.0153
SQLE	2.04	11.010	10.827	11.078	9.968	9.946	9.918	0.0041

SAMHD1	2.04	6.182	6.505	6.482	5.361	5.524	5.195	0.0019
DHX9	2.04	9.579	9.767	9.694	8.698	8.746	8.499	0.0006
SMC2	2.04	7.312	7.978	7.437	6.622	6.709	6.375	0.0229
SSX2IP	2.04	7.021	7.515	7.464	6.280	6.509	6.146	0.0082
HPGD	2.04	7.490	7.642	7.922	6.617	6.726	6.654	0.0112
LOC10065	2.04	7.376	7.439	7.404	6.490	6.142	6.476	0.0104
SPIN4	2.04	8.783	8.962	8.717	7.805	7.877	7.701	0.0006
DSCC1	2.04	8.678	8.796	8.969	7.721	7.884	7.762	0.0014
RNF128	2.04	9.981	10.356	10.358	9.179	9.447	8.983	0.0051
SYNCRIP	2.04	9.597	9.850	9.916	8.738	9.062	8.436	0.0139
JKAMP	2.04	6.639	7.119	6.991	5.867	6.031	5.807	0.0092
SFXN1	2.04	9.083	9.041	9.390	8.171	8.189	8.102	0.0085
MCM2	2.04	8.473	8.283	8.507	7.442	7.118	7.597	0.0078
SVIP	2.03	7.998	8.336	8.095	7.159	7.361	6.804	0.0094
NUP155	2.03	8.568	8.765	8.565	7.593	7.865	7.331	0.0114
SLC6A6	2.03	9.108	8.893	9.240	8.196	7.973	8.014	0.0019
KIAA0528	2.03	6.313	7.194	6.732	5.602	6.129	5.492	0.0384
TRIP13	2.03	9.530	9.567	9.807	8.544	8.581	8.723	0.0013
CNP	2.03	10.143	10.080	10.235	9.071	9.098	9.225	0.0001
BRIX1	2.03	9.016	9.338	9.235	8.130	8.399	7.985	0.0032
MGST2	2.03	10.202	10.351	10.367	9.197	9.135	9.505	0.0049
MRE11A	2.03	6.490	7.377	6.827	6.041	6.109	5.568	0.0402
SYNCRIP	2.03	7.619	8.013	7.960	6.879	7.041	6.613	0.0044
FKBP7	2.03	5.117	4.644	5.395	4.013	4.119	4.060	0.0435
SQLE	2.03	10.541	10.376	10.678	9.531	9.524	9.499	0.0068
HSPA4L	2.02	6.333	7.138	6.976	5.773	6.061	5.648	0.0382
DHCR24	2.02	9.355	8.995	9.213	8.296	7.938	8.275	0.0030
CPOX	2.02	9.349	9.203	9.453	8.407	8.283	8.277	0.0009
IDI1	2.02	9.147	9.300	9.279	8.167	8.349	8.163	0.0003
TIPIN	2.02	5.884	5.531	5.973	4.941	4.655	4.771	0.0057
NFE2L3	2.02	7.637	8.137	7.619	6.739	6.919	6.750	0.0191
ACSL3	2.02	9.694	10.067	9.783	8.881	9.102	8.481	0.0134
SMC4	2.02	7.130	8.008	7.446	6.631	6.804	6.183	0.0403
HSD17B2	2.02	7.413	7.711	7.628	6.607	6.322	6.767	0.0045
NCAPD3	2.02	10.038	9.945	10.067	9.183	8.714	9.079	0.0141
BORA	2.01	9.225	9.577	9.282	8.231	8.620	8.186	0.0052
KRR1	2.01	8.382	8.905	8.603	7.572	7.894	7.395	0.0087
RRM1	2.01	10.940	11.090	10.967	10.005	10.130	9.818	0.0022
BORA	2.01	7.117	7.481	7.295	6.280	6.368	6.238	0.0056
DEPDC1	2.01	7.152	7.827	7.203	6.628	6.550	6.002	0.0273

RAVER2	2.01	6.737	6.767	6.737	6.067	5.413	5.658	0.0321
ID1	2.01	12.045	11.838	11.658	10.826	10.522	11.134	0.0124
ABCD3	2.01	9.725	10.101	10.019	9.066	9.003	8.769	0.0028
CCDC34	2.01	7.634	7.918	7.740	6.908	6.419	6.911	0.0119
ANLN	2.01	8.105	8.681	8.632	7.499	7.728	7.195	0.0152
ATR	2.01	5.532	6.167	6.074	5.067	5.193	4.484	0.0270
FDXACB1	2.01	6.508	6.310	6.733	5.660	5.679	5.177	0.0096
SGOL2	2.00	4.110	4.639	4.512	3.532	3.561	3.179	0.0091
OPA1	2.00	7.855	8.404	8.082	7.305	7.368	6.594	0.0329
STIL	2.00	8.034	8.324	8.057	7.220	7.280	6.898	0.0032
CDCA2	2.00	6.962	7.266	7.341	5.966	6.403	6.187	0.0043
NUP107	2.00	9.926	10.285	9.971	9.100	9.280	8.781	0.0065
CSE1L	2.00	11.260	11.622	11.245	10.345	10.576	10.215	0.0038
MPZL3	0.50	5.591	5.250	5.080	6.239	6.352	6.374	0.0157
SMOX	0.50	8.148	8.093	7.559	8.899	8.784	9.161	0.0157
MTHFR	0.50	6.643	6.791	6.421	7.669	7.451	7.744	0.0023
RARA	0.50	5.736	5.695	6.041	6.956	6.702	6.833	0.0026
FAM129A	0.50	7.511	8.122	7.193	8.723	8.960	8.212	0.0456
CTAGE5	0.50	6.008	6.291	5.730	7.073	7.241	6.735	0.0104
RBMS2	0.50	9.083	9.257	8.936	9.989	10.233	10.075	0.0013
XYLT1	0.50	4.118	4.443	4.693	5.523	5.347	5.453	0.0183
MICAL2	0.50	8.143	8.281	7.866	9.031	9.208	9.094	0.0067
STK19	0.50	9.475	9.100	8.930	10.243	10.050	10.278	0.0129
GET4	0.50	8.420	8.396	8.478	9.439	9.335	9.538	0.0010
MILR1	0.50	6.550	6.841	6.521	7.593	7.434	7.898	0.0052
MXD3	0.49	8.027	7.888	7.652	8.900	8.643	9.062	0.0036
RBMS2	0.49	8.940	9.122	8.702	9.852	10.070	9.909	0.0047
MGLL	0.49	8.806	8.662	8.736	9.739	9.798	9.716	0.0001
PRSS22	0.49	9.225	9.166	9.121	10.193	10.167	10.202	0.0003
HYOU1	0.49	6.440	6.499	6.530	7.399	7.610	7.505	0.0010
HYOU1	0.49	10.080	10.061	9.961	11.020	10.981	11.150	0.0002
IL6R	0.49	3.815	3.793	3.923	4.892	5.086	4.563	0.0171
MYEOV	0.49	10.314	10.075	10.318	11.246	11.096	11.415	0.0012
MICAL2	0.49	9.454	9.579	9.192	10.436	10.557	10.303	0.0031
SH3BP4	0.49	6.106	6.330	6.199	7.364	6.995	7.317	0.0040
MXD1	0.49	7.785	7.775	7.583	8.666	8.908	8.621	0.0011
TNNT1	0.49	6.554	6.824	6.668	7.669	7.648	7.802	0.0010
SNHG8	0.49	10.538	10.646	10.334	11.516	11.545	11.540	0.0074
PRSS2	0.49	8.869	9.184	8.663	9.943	9.773	10.095	0.0076
DUSP3	0.49	7.554	7.513	7.541	8.483	8.685	8.503	0.0030
NEBL	0.49	6.167	6.477	6.270	7.332	7.479	7.179	0.0012
ACCS	0.49	6.586	6.031	6.010	7.302	7.001	7.441	0.0139

AATK	0.49	7.665	7.113	7.296	8.603	8.018	8.519	0.0143
MAP1LC3B	0.49	10.323	10.499	9.960	11.252	11.475	11.166	0.0092
TNNT1	0.49	7.475	7.456	7.374	8.538	8.225	8.596	0.0088
DUSP3	0.49	7.527	7.416	7.597	8.489	8.657	8.475	0.0002
SLFN5	0.49	4.042	4.708	3.755	5.254	5.479	4.989	0.0438
SLMO1	0.49	7.480	7.228	7.460	8.470	8.210	8.567	0.0021
MED19	0.49	9.096	9.175	9.036	10.060	10.162	10.185	0.0001
FEM1C	0.49	7.513	7.881	7.533	8.694	9.048	8.199	0.0373
ARHGAP19	0.49	3.598	3.758	3.611	4.507	5.006	4.511	0.0183
RRAGC	0.49	9.239	9.335	9.230	10.225	10.636	9.977	0.0310
TRIO	0.49	6.810	6.865	6.568	7.654	8.166	7.453	0.0272
LOC100510707	0.49	7.596	7.572	7.496	8.575	8.699	8.498	0.0006
C6orf48	0.49	11.682	11.827	11.375	12.641	12.734	12.659	0.0128
ASB1	0.49	8.813	8.770	8.717	9.708	9.693	9.996	0.0058
BCL9L	0.49	7.843	7.926	8.149	9.108	9.019	8.919	0.0016
MYEOV	0.49	8.599	8.567	8.756	9.753	9.517	9.770	0.0008
SPIRE1	0.49	7.805	8.400	7.816	8.913	9.426	8.813	0.0188
TEF	0.49	7.403	7.289	7.148	8.298	8.146	8.509	0.0020
C7orf13	0.49	7.339	7.496	7.099	8.308	8.343	8.433	0.0071
SLMO1	0.49	4.296	4.321	3.944	5.486	4.858	5.303	0.0140
SNHG1	0.49	9.858	10.037	9.790	10.792	10.937	11.079	0.0008
FLNB	0.48	10.584	10.540	10.306	11.480	11.586	11.524	0.0032
NBR2	0.48	7.138	7.511	7.286	8.404	8.392	8.309	0.0070
CCDC92	0.48	7.956	8.124	7.821	9.036	8.968	9.065	0.0036
FSTL3	0.48	4.678	4.422	5.175	5.919	5.877	5.736	0.0326
DUSP10	0.48	5.850	5.816	5.671	6.791	6.791	6.924	0.0002
C17orf51	0.48	6.224	6.127	6.158	7.385	7.146	7.133	0.0029
FHL2	0.48	11.100	11.250	10.477	12.010	12.017	12.077	0.0429
MED19	0.48	9.657	9.707	9.506	10.687	10.687	10.672	0.0031
TRIM25	0.48	5.993	6.074	5.937	7.179	6.896	7.089	0.0018
RRAGC	0.48	9.404	9.463	9.454	10.454	10.747	10.248	0.0175
GLIS2	0.48	8.672	8.554	8.515	9.659	9.563	9.693	0.0001
C1orf116	0.48	8.671	8.614	8.368	9.717	9.178	9.863	0.0238
CYTH3	0.48	8.768	8.797	8.793	9.833	9.853	9.848	0.0000
PABPC1L	0.48	8.632	8.728	8.280	9.620	9.442	9.775	0.0044
ZNFX1-AS1	0.48	12.027	12.044	11.741	12.964	13.074	12.976	0.0042
TNNT1	0.48	6.779	6.807	6.913	7.821	7.811	8.043	0.0010
COX19	0.48	6.351	6.377	6.184	7.555	7.175	7.352	0.0031
KLF4	0.48	9.890	9.864	9.632	10.928	10.672	10.972	0.0011

TMC4	0.48	8.406	8.375	7.932	9.264	9.138	9.531	0.0062
LPP	0.48	5.502	5.494	5.515	6.568	6.696	6.439	0.0046
CCRN4L	0.48	5.460	5.111	5.123	6.505	5.987	6.393	0.0070
CLDN15	0.48	6.480	6.758	6.839	7.657	7.960	7.679	0.0019
STX5	0.48	6.540	6.468	6.342	7.375	7.549	7.640	0.0006
RCAN1	0.48	6.706	6.608	6.188	7.869	7.397	7.458	0.0079
KCNJ14	0.48	5.179	5.402	5.457	6.287	6.529	6.446	0.0007
MORN4	0.47	6.349	6.464	5.965	7.296	7.234	7.504	0.0074
HYOU1	0.47	8.984	9.079	8.993	10.098	10.002	10.184	0.0003
WARS	0.47	10.756	10.962	10.416	11.780	11.891	11.749	0.0153
HSPB8	0.47	4.609	4.635	4.493	5.709	5.859	5.374	0.0119
LOC1005 06403	0.47	6.672	6.939	7.005	8.118	7.644	8.075	0.0063
RRAGC	0.47	8.967	9.071	9.088	10.058	10.357	9.938	0.0085
MORN4	0.47	5.250	5.003	4.328	6.065	5.840	6.070	0.0460
HKDC1	0.47	7.127	7.389	6.623	8.125	8.165	8.207	0.0367
SPINT1	0.47	6.870	6.682	6.674	7.836	7.845	7.813	0.0029
WARS	0.47	10.055	10.365	9.699	11.136	11.192	11.128	0.0275
WARS	0.47	9.859	10.265	9.608	10.997	11.164	10.901	0.0175
WIPI1	0.47	6.007	6.099	5.745	6.930	7.296	6.877	0.0035
C10orf54	0.47	7.432	7.353	7.420	8.391	8.499	8.576	0.0005
EIF1B	0.47	8.948	8.992	8.939	9.973	10.140	10.031	0.0008
SPIRE1	0.47	5.937	6.415	5.566	7.207	7.274	6.791	0.0253
TFE3	0.47	6.656	6.838	6.294	7.650	7.290	8.068	0.0214
C3orf52	0.47	8.824	8.996	8.683	10.042	9.791	9.951	0.0009
FHL2	0.47	10.354	10.565	9.672	11.248	11.325	11.444	0.0462
FHL2	0.47	10.886	11.025	10.217	11.749	11.872	11.916	0.0404
GABAR APL1	0.47	5.373	4.999	5.293	6.233	6.545	6.185	0.0024
MXD3	0.47	7.293	7.374	6.903	8.211	8.376	8.323	0.0105
SLC26A1 1	0.47	6.017	6.034	6.050	7.183	6.884	7.306	0.0126
RNF187	0.47	8.390	8.456	8.286	9.485	9.391	9.560	0.0001
LONP1	0.47	9.937	10.135	9.436	11.012	10.763	11.105	0.0179
U2AF1L4	0.47	6.924	6.694	6.891	7.906	7.840	8.071	0.0004
FKBP1A-SDCBP2	0.47	7.570	6.974	7.560	8.347	8.727	8.385	0.0133
TNNC1	0.47	6.605	6.694	6.448	7.555	7.592	7.897	0.0019
RRAGC	0.47	8.220	8.349	8.273	9.316	9.603	9.211	0.0068
LMCD1	0.47	9.078	9.094	9.018	10.184	9.951	10.344	0.0085
PXDC1	0.46	8.109	8.113	7.931	9.201	8.986	9.272	0.0008
SEMA3B	0.46	7.264	7.040	7.171	8.236	8.355	8.212	0.0003
NEBL	0.46	7.606	8.225	7.473	8.852	9.331	8.423	0.0354
LONP1	0.46	9.886	10.031	9.424	10.967	10.735	11.032	0.0125

LONP1	0.46	9.922	10.123	9.458	11.019	10.771	11.116	0.0144
FOSL1	0.46	7.965	7.594	7.999	9.062	8.751	9.101	0.0030
RCAN1	0.46	7.881	8.063	7.772	9.098	9.057	8.923	0.0009
C6orf48	0.46	11.611	11.763	11.272	12.622	12.722	12.709	0.0128
ZMYM5	0.46	5.191	5.053	4.495	5.979	6.369	5.799	0.0154
PNRC1	0.46	7.507	7.535	7.196	8.637	8.285	8.689	0.0028
HIST1H2AC	0.46	6.979	7.081	7.078	8.133	8.085	8.304	0.0007
RCAN1	0.46	7.862	8.125	7.720	9.154	9.072	8.906	0.0026
OSGIN1	0.45	6.507	6.263	6.371	7.481	7.507	7.571	0.0014
RBCK1	0.45	8.095	8.339	7.782	9.243	9.131	9.298	0.0130
CLIP4	0.45	4.159	3.777	3.818	5.147	5.086	4.955	0.0042
EIF1B	0.45	9.110	9.296	9.208	10.261	10.459	10.312	0.0001
MAP3K14	0.45	5.429	5.396	5.541	6.795	6.340	6.636	0.0082
HMOX1	0.45	6.559	6.572	6.906	7.732	8.024	7.734	0.0017
NABP1	0.45	7.397	7.685	7.423	8.756	8.769	8.425	0.0016
LOC392288	0.45	10.419	10.628	10.054	11.531	11.661	11.411	0.0104
GFPT1	0.45	8.785	9.525	8.590	10.025	10.563	9.842	0.0334
PPP2R5B	0.45	7.804	7.512	7.914	8.965	8.859	8.895	0.0073
SQSTM1	0.45	10.850	10.891	10.782	11.888	12.147	11.951	0.0015
CLCN4	0.45	4.512	4.856	4.980	6.171	5.779	5.889	0.0034
AK2	0.45	6.541	6.512	6.386	7.471	7.827	7.617	0.0027
SMOX	0.45	7.436	7.429	6.605	8.445	8.256	8.404	0.0432
ZMYM6	0.45	6.214	6.370	6.317	7.425	7.643	7.313	0.0021
PRSS1	0.45	7.837	8.349	7.665	9.123	8.938	9.353	0.0130
ADM2	0.44	5.856	5.587	5.360	6.887	6.530	6.911	0.0037
CCDC130	0.44	6.383	6.435	6.000	7.483	7.284	7.594	0.0034
CDKN1A	0.44	8.226	8.280	7.786	9.199	9.270	9.390	0.0101
SERPINB8	0.44	7.181	7.116	7.325	8.236	8.713	8.150	0.0140
SEC24D	0.44	6.482	6.604	6.619	7.516	8.220	7.359	0.0475
GDF15	0.44	11.837	11.670	10.907	12.663	12.607	12.842	0.0432
AKNA	0.44	4.771	5.018	4.377	6.029	5.865	5.885	0.0175
CHD2	0.44	6.313	6.856	5.933	7.666	7.783	7.312	0.0269
SLPI	0.44	9.745	9.908	9.213	10.861	10.617	11.002	0.0142
SPIRE1	0.44	7.889	8.295	7.496	9.043	9.435	8.795	0.0170
TMEM45B	0.44	5.952	6.021	5.706	7.090	7.182	6.974	0.0011
AMN1	0.44	7.180	7.462	7.022	8.299	8.652	8.281	0.0026
RIOK3	0.44	7.994	8.225	7.821	9.081	9.624	8.807	0.0243
GFPT1	0.44	9.557	10.016	9.371	10.758	11.170	10.598	0.0100

C20orf111	0.44	8.880	8.940	8.735	9.951	10.088	10.091	0.0002
MPZL3	0.44	8.930	9.025	8.744	10.218	10.128	9.924	0.0006
CDKN1A	0.44	10.023	9.961	9.390	10.984	10.996	11.054	0.0251
CLIP4	0.44	3.651	3.729	3.891	4.548	5.274	4.951	0.0219
RCAN1	0.44	7.491	7.663	7.351	8.894	8.522	8.667	0.0012
GADD45A	0.44	10.028	9.941	9.427	11.190	10.710	11.109	0.0083
AGR2	0.44	11.232	11.377	11.080	12.315	12.559	12.407	0.0005
TMEM80	0.44	6.584	6.738	6.803	8.077	7.784	7.846	0.0007
ETS1	0.44	4.754	4.398	4.610	5.947	5.599	5.813	0.0012
TCP11L2	0.43	5.107	4.947	4.239	5.960	6.111	5.971	0.0388
PNRC1	0.43	8.967	9.113	8.584	10.150	10.027	10.150	0.0121
KDM6B	0.43	4.733	5.061	4.559	6.002	5.953	6.059	0.0116
AKNA	0.43	7.047	6.885	6.829	8.132	8.027	8.222	0.0002
EPHX2	0.43	4.885	4.972	4.732	5.901	6.179	6.131	0.0005
PLCXD2	0.43	6.953	7.434	6.533	8.496	7.843	8.275	0.0221
TNFRSF12A	0.43	11.243	11.129	11.024	12.303	12.178	12.531	0.0014
LAMB3	0.43	8.077	7.995	7.814	9.222	9.364	8.907	0.0036
SEMA3B	0.43	6.768	6.221	6.364	7.643	7.709	7.691	0.0162
HDAC9	0.43	5.480	5.985	5.715	6.843	7.291	6.648	0.0088
EXD3	0.43	6.042	6.210	5.832	7.128	7.307	7.302	0.0021
NBR2	0.43	6.358	6.959	6.268	7.562	7.830	7.911	0.0155
CREB3L2	0.43	5.998	5.903	5.652	7.127	6.978	7.109	0.0023
ZNF185	0.43	5.540	5.639	5.912	6.870	7.130	6.738	0.0016
KDM6B	0.43	4.623	4.990	4.507	5.924	5.833	6.059	0.0059
SERPINB8	0.43	6.105	6.499	5.925	7.325	7.583	7.329	0.0078
RNF187	0.43	5.542	5.578	5.222	6.580	6.625	6.815	0.0017
RELB	0.43	7.762	7.821	7.682	8.914	8.999	9.018	0.0000
ITPKC	0.43	7.118	6.885	6.634	8.216	7.745	8.325	0.0067
HERC4	0.43	5.555	5.616	5.521	6.960	6.715	6.683	0.0026
INPP4B	0.43	5.360	4.623	5.690	6.416	6.734	6.394	0.0428
GJB3	0.43	8.502	8.345	8.558	9.702	9.637	9.773	0.0003
AMOTL2	0.42	7.782	7.803	7.476	9.112	8.547	9.068	0.0083
ZMYM5	0.42	7.071	7.352	6.847	8.183	8.631	8.166	0.0043
CARD9	0.42	4.089	4.177	4.318	5.779	5.065	5.383	0.0197
HMOX1	0.42	7.407	7.407	7.483	8.549	8.978	8.457	0.0149
YPEL5	0.42	7.245	7.790	7.166	8.502	9.043	8.407	0.0110
GDF15	0.42	11.768	11.595	10.783	12.594	12.573	12.903	0.0381
RIOK3	0.42	9.934	10.302	9.733	11.170	11.609	10.940	0.0089
ELL2	0.42	6.398	6.623	6.300	7.782	7.658	7.671	0.0021

EGR2	0.42	4.932	4.403	4.343	5.897	5.392	6.153	0.0134
ABHD4	0.41	7.318	7.435	7.023	8.502	8.465	8.643	0.0035
FOSL1	0.41	7.353	7.052	7.396	8.620	8.141	8.809	0.0106
GOLT1A	0.41	9.125	9.213	8.891	10.190	10.588	10.267	0.0015
VIMP	0.41	6.789	6.962	6.590	7.966	8.206	8.021	0.0011
CREB3L2	0.41	5.974	5.900	5.568	7.206	6.915	7.181	0.0016
MAP1LC3B	0.41	9.715	9.853	9.112	10.930	10.966	10.721	0.0200
NA	0.41	4.174	3.751	4.047	5.404	5.558	4.781	0.0180
C17orf110	0.41	6.381	6.509	6.306	7.371	7.787	7.853	0.0070
HMOX1	0.41	8.286	8.286	8.336	9.334	9.913	9.459	0.0180
GDF15	0.41	11.258	11.096	10.261	12.122	12.095	12.424	0.0378
MPZL3	0.41	6.129	6.115	5.966	7.325	7.339	7.434	0.0001
CREBRF	0.41	3.870	4.517	3.799	5.075	5.951	4.957	0.0354
LGALS4	0.41	9.383	9.690	8.843	10.642	10.514	10.774	0.0246
FKBP14	0.41	5.767	6.234	5.893	7.197	7.526	7.086	0.0025
PARD6B	0.40	8.092	8.385	8.179	9.576	9.443	9.565	0.0010
FOSL1	0.40	7.341	6.947	7.436	8.708	8.145	8.766	0.0078
NABP1	0.40	6.788	6.976	6.864	8.285	8.142	8.146	0.0001
MEF2D	0.40	8.101	8.173	7.656	9.391	8.972	9.510	0.0046
C17orf110	0.40	6.499	6.793	6.035	7.670	7.837	7.865	0.0197
ELL2	0.40	5.892	6.149	5.787	7.290	7.333	7.178	0.0023
LAMB3	0.40	10.180	10.209	9.800	11.394	11.415	11.373	0.0092
AGR2	0.40	9.053	9.329	8.922	10.240	10.529	10.518	0.0012
RIOK3	0.40	8.383	8.719	7.997	9.593	10.112	9.358	0.0124
RBCK1	0.40	8.721	8.899	8.247	10.010	9.836	10.079	0.0120
RIOK3	0.40	8.783	9.230	8.636	10.036	10.687	9.852	0.0168
CHIC2	0.40	8.855	8.900	8.579	10.267	10.077	10.003	0.0006
TMEM184A	0.40	5.107	5.360	5.321	6.735	6.478	6.591	0.0003
BTG1	0.40	9.755	9.810	8.818	10.922	10.861	10.810	0.0476
PRKAB2	0.39	7.125	7.584	7.200	8.719	8.822	8.414	0.0021
BTG1	0.39	8.910	9.047	8.081	10.108	10.081	10.080	0.0428
HKDC1	0.39	9.864	10.176	9.152	11.153	11.216	11.059	0.0405
GEM	0.39	4.184	4.817	4.069	5.626	6.108	5.430	0.0119
ARMCX3	0.39	8.082	8.466	8.266	9.546	10.055	9.162	0.0230
AXL	0.39	5.951	5.715	6.004	7.185	7.463	7.077	0.0010
C17orf110	0.39	7.057	7.244	6.776	8.270	8.452	8.461	0.0036
ARMCX3	0.39	7.608	8.103	7.753	9.150	9.577	8.742	0.0144
ELL2	0.39	5.210	5.710	5.433	6.809	6.805	6.864	0.0099

LETM2	0.39	8.301	8.414	8.288	9.600	9.722	9.766	0.0000
ARID3B	0.39	5.826	5.776	5.927	7.171	7.332	7.120	0.0001
PTPRH	0.39	8.416	8.471	7.834	9.638	9.493	9.763	0.0122
C4BPB	0.39	5.620	5.501	6.194	7.315	7.270	6.911	0.0090
DUSP1	0.39	5.756	5.178	5.253	6.957	6.595	6.809	0.0056
SCEL	0.39	5.364	6.513	5.644	7.162	7.768	6.813	0.0361
SCEL	0.38	6.676	7.572	7.126	8.314	9.031	8.144	0.0218
MAP1LC3B	0.38	8.422	8.537	7.771	9.764	9.670	9.539	0.0212
LURAP1L	0.38	8.077	8.213	7.065	9.416	8.984	9.314	0.0440
FOSL1	0.38	8.671	8.518	8.749	10.163	9.746	10.153	0.0032
FKBP14	0.38	5.336	5.562	5.379	6.999	6.968	6.411	0.0115
MKNK2	0.38	8.848	9.167	8.848	10.316	10.529	10.198	0.0007
FKBP1A-SDCBP2	0.38	7.166	7.214	7.270	8.402	8.862	8.544	0.0073
C1orf116	0.38	8.302	8.257	7.964	9.571	9.393	9.774	0.0008
RBCK1	0.37	8.241	8.622	7.882	9.806	9.511	9.764	0.0112
ABHD4	0.37	5.546	5.728	5.417	7.118	6.866	6.977	0.0003
DUSP1	0.37	5.858	5.374	5.400	7.170	6.528	7.177	0.0077
LAMP3	0.37	4.370	4.572	4.633	5.873	6.306	5.616	0.0112
KRT7	0.37	8.067	8.297	7.948	9.463	9.639	9.542	0.0012
MAP3K14	0.37	7.070	6.853	6.690	8.538	7.696	8.570	0.0272
ELL2	0.37	6.785	7.352	7.148	8.653	8.698	8.285	0.0028
NABP1	0.37	7.321	7.616	7.315	9.018	8.893	8.689	0.0005
GTPBP2	0.37	8.939	9.121	7.995	10.298	9.904	10.397	0.0339
LOC100509764	0.36	6.661	6.671	6.542	8.069	7.960	8.213	0.0003
EPB41L4A-AS1	0.36	10.447	10.648	10.094	11.795	11.931	11.912	0.0083
PER1	0.36	5.064	4.634	5.086	6.483	5.988	6.698	0.0065
FOXA3	0.36	5.867	5.725	5.167	7.281	6.889	7.120	0.0079
YPEL5	0.36	8.630	9.047	8.292	10.137	10.408	9.960	0.0074
MAP2	0.35	5.155	5.324	5.849	6.709	7.282	6.854	0.0057
PTPRH	0.35	6.431	6.839	5.818	7.921	7.837	7.998	0.0318
SCEL	0.35	6.433	7.442	6.663	8.224	8.885	7.969	0.0214
C10orf54	0.35	5.176	5.286	5.115	6.941	6.737	6.366	0.0082
MIR22	0.35	5.592	5.924	5.692	7.213	7.166	7.369	0.0005
SCEL	0.35	5.659	6.691	5.826	7.538	8.094	7.166	0.0222
ABHD4	0.35	5.698	5.564	5.679	7.146	7.221	7.128	0.0000
GTPBP2	0.35	6.609	6.505	5.728	8.041	7.551	7.910	0.0155
DUSP10	0.35	4.300	4.501	4.274	5.810	5.791	6.035	0.0001
DUSP10	0.35	5.331	4.890	4.682	6.425	6.396	6.704	0.0057

NBR2	0.35	6.270	6.681	5.489	7.705	7.769	7.789	0.0435
LCN2	0.35	11.134	11.503	10.796	12.647	12.767	12.691	0.0147
MIR22	0.35	6.838	7.047	6.922	8.524	8.422	8.470	0.0002
JUN	0.35	9.479	9.329	9.488	11.139	10.485	11.181	0.0177
CSRNP1	0.35	7.694	7.382	7.475	9.086	8.614	9.374	0.0113
SCEL	0.34	5.451	6.271	5.709	7.298	7.803	6.931	0.0119
SCEL	0.34	5.867	7.008	6.016	7.774	8.319	7.589	0.0268
MAP2	0.34	4.570	4.965	4.674	6.254	6.768	5.601	0.0372
TMEM217	0.34	5.197	4.461	4.834	6.401	6.144	6.620	0.0058
KLF6	0.34	8.761	8.663	8.521	10.322	10.196	10.098	0.0001
JUN	0.34	7.842	7.771	7.893	9.612	8.914	9.565	0.0190
ARID3B	0.34	7.976	8.004	8.293	9.613	9.741	9.650	0.0016
DNAJB9	0.34	8.308	9.102	8.355	10.150	10.414	10.033	0.0134
PRKAB2	0.33	5.415	5.441	5.335	6.867	7.453	6.455	0.0328
KLF6	0.33	8.933	8.903	8.768	10.568	10.488	10.327	0.0001
GABARAPL1	0.33	6.481	6.423	5.876	7.815	7.909	7.917	0.0120
FKBP14	0.33	4.768	5.137	4.798	6.483	6.816	6.146	0.0044
JUN	0.33	8.596	8.361	8.496	10.225	9.717	10.254	0.0059
CLIP4	0.33	5.805	5.882	5.503	7.588	7.355	7.059	0.0015
JUN	0.32	8.532	8.411	8.510	10.322	9.664	10.299	0.0153
LINC00341	0.32	5.113	5.193	4.378	6.730	6.355	6.649	0.0122
C2orf54	0.32	5.096	5.114	5.207	6.570	7.209	6.478	0.0179
CLDN1	0.32	9.014	9.313	8.103	10.688	10.448	10.508	0.0368
AIM1L	0.32	7.200	7.261	6.920	8.781	8.571	8.993	0.0006
CLDN1	0.32	9.395	9.628	8.528	11.032	10.850	10.871	0.0322
KLF6	0.31	7.037	6.866	6.743	8.700	8.606	8.345	0.0003
MIR22	0.31	5.867	6.399	5.885	7.838	7.633	7.761	0.0058
ARG2	0.31	7.543	8.026	7.026	9.178	9.468	9.123	0.0173
MIR22	0.31	6.506	7.033	6.734	8.569	8.336	8.443	0.0029
ZFAND2A	0.31	9.260	9.304	9.228	10.800	11.006	11.020	0.0008
CST6	0.31	6.123	6.041	6.548	7.657	8.270	7.824	0.0024
DUSP5	0.31	9.089	9.135	8.809	10.732	10.561	10.817	0.0003
CLDN1	0.31	6.926	6.838	6.040	8.433	8.241	8.350	0.0218
NIT1	0.31	5.592	5.159	5.102	6.992	6.985	7.008	0.0080
DUSP8	0.31	5.535	5.392	5.215	7.092	6.383	7.555	0.0337
UPP1	0.31	10.321	10.347	10.186	11.901	12.151	11.917	0.0002
PPP1R15A	0.30	8.390	8.364	7.833	9.970	9.823	10.001	0.0067
AGR2	0.30	7.517	8.014	7.119	9.153	9.602	9.138	0.0082
HSPB8	0.30	4.128	4.401	4.139	5.725	6.241	5.836	0.0020

DNAJB9	0.30	6.694	7.732	6.625	8.570	9.224	8.621	0.0198
SQSTM1	0.30	9.297	9.327	9.271	10.834	11.282	10.986	0.0052
CLDN1	0.29	7.639	7.901	6.910	9.403	9.231	9.331	0.0226
KRT80	0.29	9.206	9.118	9.085	10.934	10.845	11.023	0.0000
DUSP8	0.28	5.598	5.642	5.558	7.518	6.794	7.757	0.0254
ISG20	0.28	5.465	5.654	5.145	7.119	7.468	7.144	0.0009
UPP1	0.28	9.023	9.275	9.076	10.773	11.239	10.876	0.0013
ARG2	0.28	6.888	7.392	6.231	8.652	8.898	8.742	0.0249
PRSS1	0.27	7.093	7.964	7.324	9.329	9.307	9.577	0.0111
PEAR1	0.26	4.527	4.672	4.486	6.642	6.080	6.684	0.0066
ETS1	0.26	5.979	5.624	6.240	8.021	7.954	7.765	0.0031
MAFF	0.26	7.213	7.141	6.785	9.109	8.581	9.285	0.0029
CYR61	0.26	7.429	7.000	7.207	9.470	8.264	9.518	0.0358
CYR61	0.23	7.424	7.101	7.195	9.582	8.419	9.794	0.0364
ETS1	0.22	8.839	8.883	8.825	11.133	11.075	10.828	0.0014
IL20RB	0.21	3.877	4.433	3.642	6.533	5.909	6.231	0.0021
CTGF	0.20	6.484	6.003	5.362	8.681	7.108	8.678	0.0313
ATF3	0.20	6.611	6.053	5.673	8.743	7.536	8.863	0.0155
ATF3	0.18	7.412	7.155	6.339	9.607	8.266	10.032	0.0282
ATF3	0.18	8.739	8.586	7.651	11.042	9.821	11.281	0.0157
ATF3	0.17	8.087	7.889	6.915	10.373	9.192	10.723	0.0155
LAMP3	0.16	5.645	6.130	5.558	8.189	8.835	8.227	0.0007
DUSP1	0.15	6.512	6.332	5.962	9.228	8.194	9.297	0.0084
DUSP1	0.13	6.204	6.492	5.874	9.041	8.472	9.618	0.0044

Note: GS= Gene Symbol, FC= Fold change, T-1= ZLD1039_Treat_1, CTL-1= Control_1, the differental genes were filtered by fold-change > 2 and *P* value < 0.05.
